# Use of Digital Health Technologies for Dementia Care: Bibliometric Analysis and Report

**DOI:** 10.2196/64445

**Published:** 2025-02-10

**Authors:** Hebatullah Abdulazeem, Israel Júnior Borges do Nascimento, Ishanka Weerasekara, Amin Sharifan, Victor Grandi Bianco, Ciara Cunningham, Indunil Kularathne, Genevieve Deeken, Jerome de Barros, Brijesh Sathian, Lasse Østengaard, Frederique Lamontagne-Godwin, Joost van Hoof, Ledia Lazeri, Cassie Redlich, Hannah R Marston, Ryan Alistair Dos Santos, Natasha Azzopardi-Muscat, Yongjie Yon, David Novillo-Ortiz

**Affiliations:** 1 Division of Country Health Policies and Systems World Health Organization Regional Office for Europe Copenhagen Denmark; 2 Technical University of Munich TUM School of Medicine and Heath Munich Germany; 3 Center for Research in Epidemiology and Statistics (CRESS-U1153), Université Paris Cité and Université Sorbonne Paris Nord, INRAE Hôpital Hôtel-Dieu Paris France; 4 School of Medicine, Faculdade de Ciências Médicas de Minas Gerais Minas Gerais Brazil; 5 Faculty of Health and Social Sciences Western Norway University of Applied Sciences Bergen Norway; 6 School of Health Sciences, The University of Newcastle Callaghan Australia; 7 Institute of Health and Wellbeing, Federation University Australia Melbourne Australia; 8 Department for Evidence-based Medicine and Evaluation, University for Continuing Education Krems Krems an der Donau Austria; 9 Rio de Janeiro State University (UERJ) Núcleo de Estudos e Pesquisas em Atenção ao Uso de Drogas (NEPAD) Rio de Janeiro Brazil; 10 Sports Medicine Unit National Hospital of Kandy Kandy Sri Lanka; 11 Center for Research in Epidemiology and Statistics (CRESS-U1153), Université Paris Cité and Université Sorbonne Paris Nord, Inserm, Epidemiology of Childhood and Adolescent Cancers (EPICEA) Hôpital Paul Brosse AP-HP Villejuif France; 12 Directorate-General for Health and Food Safety, European Commission Brussels Belgium; 13 Geriatrics and Long-Term Care Department Rumailah Hospital, Hamad Medical Corporation Doha Qatar; 14 Centre for Evidence-Based Medicine Odense (CEBMO) and Cochrane Denmark, Department of Clinical Research University of Southern Denmark Odense Denmark; 15 Research Group of Urban Ageing, Faculty of Social Work and Education The Hague University of Applied Sciences The Hague Netherlands; 16 Faculty of Spatial Management and Landscape Architecture, Department of Systems Research, Wrocław University of Environmental and Life Sciences Wrocław Poland; 17 School of Health, Wellbeing and Social Care The Open University Milton Keynes United Kingdom

**Keywords:** people living with dementia, digital health technologies, bibliometric analysis, evidence-based medicine

## Abstract

**Background:**

Dementia is a syndrome that compromises neurocognitive functions of the individual and that is affecting 55 million individuals globally, as well as global health care systems, national economic systems, and family members.

**Objective:**

This study aimed to determine the status quo of scientific production on use of digital health technologies (DHTs) to support (older) people living with dementia, their families, and care partners. In addition, our study aimed to map the current landscape of global research initiatives on DHTs on the prevention, diagnosis, treatment, and support of people living with dementia and their caregivers.

**Methods:**

A bibliometric analysis was performed as part of a systematic review protocol using MEDLINE, Embase, Scopus, Epistemonikos, the Cochrane Database of Systematic Reviews, and Google Scholar for systematic and scoping reviews on DHTs and dementia up to February 21, 2024. Search terms included various forms of dementia and DHTs. Two independent reviewers conducted a 2-stage screening process with disagreements resolved by a third reviewer. Eligible reviews were then subjected to a bibliometric analysis using VOSviewer to evaluate document types, authorship, countries, institutions, journal sources, references, and keywords, creating social network maps to visualize emergent research trends.

**Results:**

A total of 704 records met the inclusion criteria for bibliometric analysis. Most reviews were systematic, with a substantial number covering mobile health, telehealth, and computer-based cognitive interventions. Bibliometric analysis revealed that the *Journal of Medical Internet Research* had the highest number of reviews and citations. Researchers from 66 countries contributed, with the United Kingdom and the United States as the most prolific. Overall, the number of publications covering the intersection of DHTs and dementia has increased steadily over time. However, the diversity of reviews conducted on a single topic has resulted in duplicated scientific efforts. Our assessment of contributions from countries, institutions, and key stakeholders reveals significant trends and knowledge gaps, particularly highlighting the dominance of high-income countries in this research domain. Furthermore, our findings emphasize the critical importance of interdisciplinary, collaborative teams and offer clear directions for future research, especially in underrepresented regions.

**Conclusions:**

Our study shows a steady increase in dementia- and DHT-related publications, particularly in areas such as mobile health, virtual reality, artificial intelligence, and sensor-based technologies interventions. This increase underscores the importance of systematic approaches and interdisciplinary collaborations, while identifying knowledge gaps, especially in lower-income regions. It is crucial that researchers worldwide adhere to evidence-based medicine principles to avoid duplication of efforts. This analysis offers a valuable foundation for policy makers and academics, emphasizing the need for an international collaborative task force to address knowledge gaps and advance dementia care globally.

**Trial Registration:**

PROSPERO CRD42024511241; https://www.crd.york.ac.uk/prospero/display_record.php?RecordID=511241

## Introduction

### Background

Dementia is a progressive neurocognitive syndrome currently cited as the seventh leading cause of death worldwide [1]. This degenerative disease is also one of the leading causes of disability or dependency for aging populations today [2]. Dementia places a substantial burden on health care systems, national economies, and families, who often bear the responsibility of caregiving. Beyond the financial and logistical strain, individuals with dementia endure profound cognitive and neurological challenges, significantly affecting their quality of life [3]. In 2019, dementia affected over 55 million people globally and cost the world economy US $1.3 trillion. Nearly half of the financial burden was borne by informal caregivers [4]. With no currently available cure [5], efforts are focused on disease management, improving quality of life, and providing well-being support [6].

In recent years, the health care landscape has undergone a profound transformation with the integration of digital health technologies (DHTs), particularly the technologies applicable in the care of people living with dementia and their health care providers (formal and informal). Digital solutions offer novel avenues for enhancing medical care by supporting early diagnoses and implementing better preventive strategies [7]. Furthermore, DHTs can potentially minimize the burden of care felt by family members, who often devote over 5 hours daily to providing care and supervision [8]. Digital health interventions can offer a multifaceted approach to dementia treatment and prevention and revolutionize the modern approach to health care delivery and support in daily living. This comprehensive group of interventions covers multiple modalities of technologies, including artificial intelligence (AI), computer-based cognitive interventions and digital platforms, GPS, sensors for remote care, smart devices, mobile health, instant messaging applications, wearable devices, virtual reality, and telehealth [9].

Recognizing the value of multidisciplinary research initiatives, the World Health Organization (WHO) Regional Office for Europe has taken a leading role in advancing scientific research in this area. Key initiatives have made significant contributions to exploring the intersection between digital health and healthy aging, including the United Nations Decade of Healthy Ageing (2021-2030) [10], the WHO Global Digital Health Strategy 2020 to 2025, the Regional Digital Health action plan for the WHO European Region 2023 to 2030, and the WHO European Framework for Action on Mental Health 2021 to 2025. Acknowledging the importance of its role, the WHO is committed to supporting the achievement of the sustainable development goals, specifically goal 3 (good health and well-being) and goal 9 (industry, innovation, and infrastructure) [11]. The research questions in this study aligned with 4 particular targets: reduce mortality from noncommunicable diseases and promote mental health (3.4), achieve universal health coverage (3.8), enhance research and upgrade industrial technologies (9.5), and universal access to information and communications technology (9.8) [11].

A protocol was published on February 19, 2024, which provided an overview of systematic and scoping reviews detailing how the analysis of different modalities of DHTs could improve the prevention, diagnosis, treatment, care, and support of (older) people living with dementia, their families, wider support network, and care partners [12]. The research insights were drawn from a series of reviews on the topic and the numerous interventions for the holistic management of dementia, underscoring the necessity for compiling and summarizing the existing evidence in a solid and systematic document. As of April 2024, there has been a paucity of comprehensive, high-quality bibliometric analysis that examines the complete scope of research intersecting between the interface of DHTs and dementia care. Notably, compiling this bibliometric analysis provides valuable insights to identify areas for further investigation in granular detail, acting as a conduit for researchers working in areas of technology, gerontology, social science, gerontechnology, social research, and policy to initiate a route map based on the gaps in the literature.

### This Review

This publication synthesizes the preliminary findings from the broad systematic search on February 21, 2024. This work will serve as a springboard for future investigations with a more detailed synthesis of specific modalities in digital health and how they relate to the 8 domains of the WHO’s Age-friendly Cities and Communities Framework. We anticipate that this series of documents will yield evidence beneficial to clinicians and policy makers alike working with people living with dementia.

## Methods

### Overview

Our study combines 2 methodologies within the field of evidence-based medicine as follows: (1) overview of systematic reviews and (2) bibliometric analysis. We combined both methodologies to leverage their complementary strengths in analyzing the complexity and evolution of DHTs in dementia care [13]. We initially used the principles of systematic reviews to obtain a broader number of studies, enabling the execution of the bibliometric analysis. The integration of both approaches provided a comprehensive and in-depth understanding of the current state of DHTs in dementia care—insights that neither method could achieve alone. This combined approach ensured rigorous and replicable evaluation of existing evidence from systematic reviews and also positioned our research within the broader context of global research trends.

This bibliometric analysis originated as part of the protocol published before the start of the reviewing process under the PROSPERO tracking locator CRD42024511241 [12]. For this bibliometric analysis, we adhered to the guidelines for bibliometric studies, BIBLIO statement [14] and report the associated checklist as Multimedia Appendix 1. This analysis began with a large-scale review of 4839 articles retrieved from a comprehensive overview of systematic reviews, examining the current literature landscape at the interface of digital health and dementia. According to the international guidelines in evidence-based medicine, overviews of reviews are intended to evaluate the existing evidence from 2 or more reviews of different health interventions for a similar population or condition; they also assess these interventions across varying outcomes, settings, or time points [15].

### Article Search and Selection Strategy

This publication is a part of the project protocol using 2 primary techniques—systematic review and bibliometric analysis. In the first phase, we systematically searched 5 leading medical databases (MEDLINE, Embase, Scopus, Epistemonikos, and Cochrane Database of Systematic Reviews) for eligible systematic and scoping reviews. In collaboration with an expert committee, our information specialist designed a search strategy based on Medical Subject Headings and nonstandardized technical terms. In addition, the search was complemented by obtaining the first 300 hits from the Scholar (Google LLC) platform (source for gray literature) for eligible reviews from database inception until February 21, 2024, regardless of publication language. Search terms involved, but were not limited to, “dementia,” “Alzheimer disease,” “Lewy body dementia,” “mixed dementias,” “Huntington disease,” “multi-infarct dementia,” “vascular dementia,” and “major neurocognitive disorder.” Full access to the search strategy used in these preliminary findings is available in Multimedia Appendix 2.

Most investigators participated in the study selection phase, which was conducted independently by at least 2 investigators using the Covidence Systematic Review Platform (Veritas Health Innovation). The screening process was conducted in two stages as follows: (1) paper title and abstract and (2) full-text screening. Disagreements were resolved by a third investigator not involved in the initial screening process by evaluating the raised discrepancies compared to the inclusion criteria in the published protocol. Interrater reliability was noted to be average with a mean (SD) of 0.66 (0.31) and 0.57 (0.28) and median (IQR) of 0.58 (0.48-0.79) and 0.66 (0.54-0.77) for the screening stages, respectively, indicating moderate to strong reliability.

Using the PICOS framework, the search criteria for review analysis are described below.

- Participants (P): reviews, including data from patients diagnosed with dementia (self-reported or clinically diagnosed by an individual or multidisciplinary team of medical and health care providers), regardless of the international classification used (for instance, *International Classification of Diseases, Tenth Revision*; *International Classification of Diseases, Eleventh Revision*; or *Diagnostic and Statistical Manual of Mental Disorders, Fifth Edition*). In addition, those reviews focusing on the importance of DHTs for caregivers (formal or informal) of people living with dementia were shortlisted and evaluated.
- Intervention (I): we included any modality of DHTs, telemedicine, telehealth, computerized decision support systems, clinical reminders or alert systems, home automation and monitoring systems, sensor-based systems and ambient intelligence, AI, mobile health, big data, 4G or 5G, exergaming, and the internet of things.
- Comparator (C): no comparison group was prioritized. We included studies without any comparator, head-to-head comparisons, and placebo assessments.
- Outcome (O): the Crosslingual Optimized Metric for Evaluation of Translation initiative was used to evaluate the existing “core outcome sets.”
- Study design (S): systematic, rapid, and scoping reviews.

Systematic reviews that included more than one database search, in either narrative or quantitative format, were eligible. Moreover, scoping and rapid reviews were considered, categorized as “a review aiming to identify and map the available evidence, but that utilized a systematic methodological approach for including studies” [16]. We excluded

narrative and integrative reviews (reviews without systematization of search and inclusion approaches) because they are more likely to report data in a highly biased pattern. Regarding the exclusion of narrative and integrative reviews, we would like to highlight several reasons [16,17]. First, narrative reviews are highly prone to selection bias, as they typically do not systematically cover all relevant studies. Similarly, integrative reviews often lack the rigorous, standardized search and inclusion criteria that are fundamental to systematic and scoping reviews [16,17]. Therefore, we excluded these types of publications to minimize potential inconsistencies and biases that could compromise the validity of our bibliometric analysis. [16,17].

### Bibliometric Analysis and Data Management

Following the initial identification of eligible reviews, a bibliometric analysis was conducted on the records that met our inclusion criterion. First, we downloaded eligible data from Covidence in a cvs format file to create our “primary library.” Raw cvs data were transformed into a relational Research Information System database, with the further exclusion of duplicates not primarily identified by Covidence (“secondary library”). After identifying unique records using their digital object identifier (DOIs), we created a matching “secondary library.” We then downloaded a comprehensive cvs file, which was further used for the bibliometric analyses.

This review used VOSviewer (version 1.6.20; Leiden University) to analyze the type of documents, years, authors, countries, institutions, journal sources, references, and keywords, allowing the creation of social network maps. VOSviewer uses probabilistic-driven normalization to ensure that the strengthen of relationships between nodes is accurately represented, accounting for differences in publication volumes and citations practices across various fields [18]. The projected clusters, each represented by a different color, suggest groups of related items that frequently cooccur in the literature, indicating underlying thematic connections [18]. The importance of these clusters lies in their ability to identify trending research topics, emerging trends, and potential gaps in the literature [18]. The total number of citations each year was obtained from the Scopus citation report. Social network analysis was set as the primary method used to analyze co-occurrence. Data associated with the journal’s impact factor (IF) were obtained from the Clarivate Journal Citation Reports (2022) and matched to the highlighted journals in our results section. From all variables prioritized in our study, corresponding data obtained from VOSviewer visualization analysis and calculations were also exported and tabulated on Microsoft Excel. VOSviewer uses probabilistic-based data normalization to create maps in fields like keywords, countries, and authors. These data were subsequently displayed in descriptive tables.

In bibliometrics, social network assessments are widely used to identify research hot spots and trends within specific scientific fields. The cluster findings were evaluated through VOSviewer to develop social network visualizations, demonstrating the relevance of the node size and line thickness. The nodes represent the number of occurrences or frequencies, while the observed lines between the nodes suggest relationships between nodes. A thicker line denotes a more substantial relationship between the nodal components, while a slimmer line denotes a weaker relationship and connection between nodes. The reported network analyses afford the visualization of trends in reviews on the interface between digital health and dementia.

Data management was performed predominantly in VOSviewer, which uses machine learning algorithms, processed input data related to study identification (title, authors, publication year, journal, and DOI), the number of citations and impact (citation count and IF of the journal in which the review was published), reported geographic regions attributed during indexation, departments and institutions declared during indexation, as well as main descriptors (standardized or nonstandardized indexation nomenclature).

Our bibliometric analysis is based on an overview of systematic and scoping reviews. Bibliometric analyses focus on quantifying publication patterns within a field, including the number of citations, authorships, coauthorship networks, institutional contributions, and keyword trends. Consequently, readers should not expect quality assessments of individual reviews, such as the use of AMSTAR tool.

### Additional Data Processing and Categorization and Data Synthesis

In addition to our bibliometric assessment, a further classification of included reviews using a 3-step data assessment plan was conducted independently using Covidence. Each review was tagged for the number of primary studies reported in the manuscript (for instance, 0-10, 11-30, 31-60, 61-90, and 91-infinity), the modality of DHT being reported within the review, and if the record was a “systematic” or “scoping” review. We designed a focused analytic step of shortlisted studies to identify overviews intersecting between digital health and dementia. An additional filtering step was applied to identify records that had already used an overview methodology in their assessment. This step was introduced to avoid duplicating research efforts and to prioritize a more detailed exploration of modalities that have historically been less examined in the literature. This resulted in the identification of 14 records with the identifier “overview” or “umbrella,” as shown in Multimedia Appendix 3.

### Ethical Considerations

Ethics approval was waived as we used secondary, publicly available data.

## Results

### Overview

**Figure 1:**
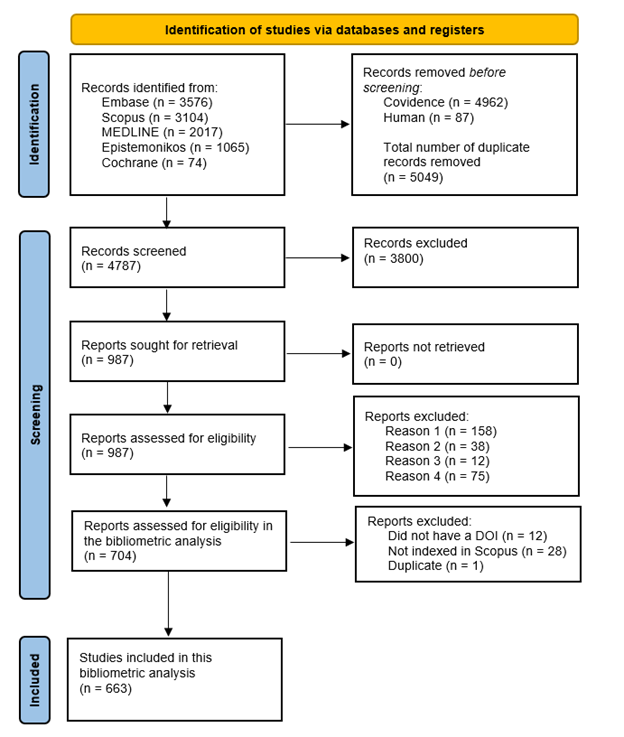
Review flowchart diagram. DOI: digital object identifier.

A review flowchart diagram is shown in Figure 1. Complete details regarding the justification for the exclusion of the shortlisted studies is available in Multimedia Appendix 4. Reasons for excluding shortlisted studies were primarily associated with conference papers (not published as full reports), protocols, reviews not focusing on DHTs, reviews of bioinformatics concepts, literature reviews, and studies not enrolling patients with dementia or cognitive impairment.

### General Findings

Our results show the categorization of eligible reviews based on the number of primary studies, the modalities of DHTs reported within reviews, and the type of review (scoping or systematic review). Most of the included records were systematic reviews (537/663, 80.9%), while some scoping reviews were also registered (126/663, 19%). In addition, most reviews (346/663, 52.1%) included 11 to 30 primary studies (regardless of the study design). Notably, a lower number of records were allocated to the 31 to 60, 61 to 90, and ≥91 primary studies.

At the time of conducting the literature search, we ascertained that there were no published protocols of an overview of reviews (systematic or nonsystematic reviews), collating the available evidence of multiple modalities of DHTs pertaining to the integrated care of people living with dementia or any other related neurocognitive disorders or cognitive impairment disorders. This literature search is significant because of the volume of studies involved, including a substantial number of published overviews analyzing the relevance of DHTs in dementia care. While we assessed DHT modalities already covered in existing literature reviews, we placed particular emphasis on thoroughly examined less-explored modalities using our overview methodology. In doing so, we ensured a focused and detailed exploration of underrepresented areas. As part of this process, 14 records containing the term “overview” or “umbrella review” were identified in the tracked records (Multimedia Appendix 3). These overviews, published after 2022, have been undertaken on people diagnosed with dementia to understand the effect of DHTs, such as wearable sensors, AI, virtual reality, eHealth, and web-based interventions. We did not include studies without a clear clinical, radiological, or any other deterministic form of diagnosing dementia on the patients under investigation in our analysis.

### Bibliometric Analyses

A total of 755 references were included, and 52 reviews were duplicated (not primarily identified through automatic exclusion using the reference manager software), resulting in 704 references eligible for the bibliometric analysis. Furthermore, 12 references did not have a DOI identifier (Multimedia Appendix 5), and 28 were not indexed in Scopus (Multimedia Appendix 6). Therefore, a total of 663 articles were downloaded into the cvs extension from Scopus, and visualization analysis was conducted.

### Top Contributing and Co-Cited Journals

**Figure 2:**
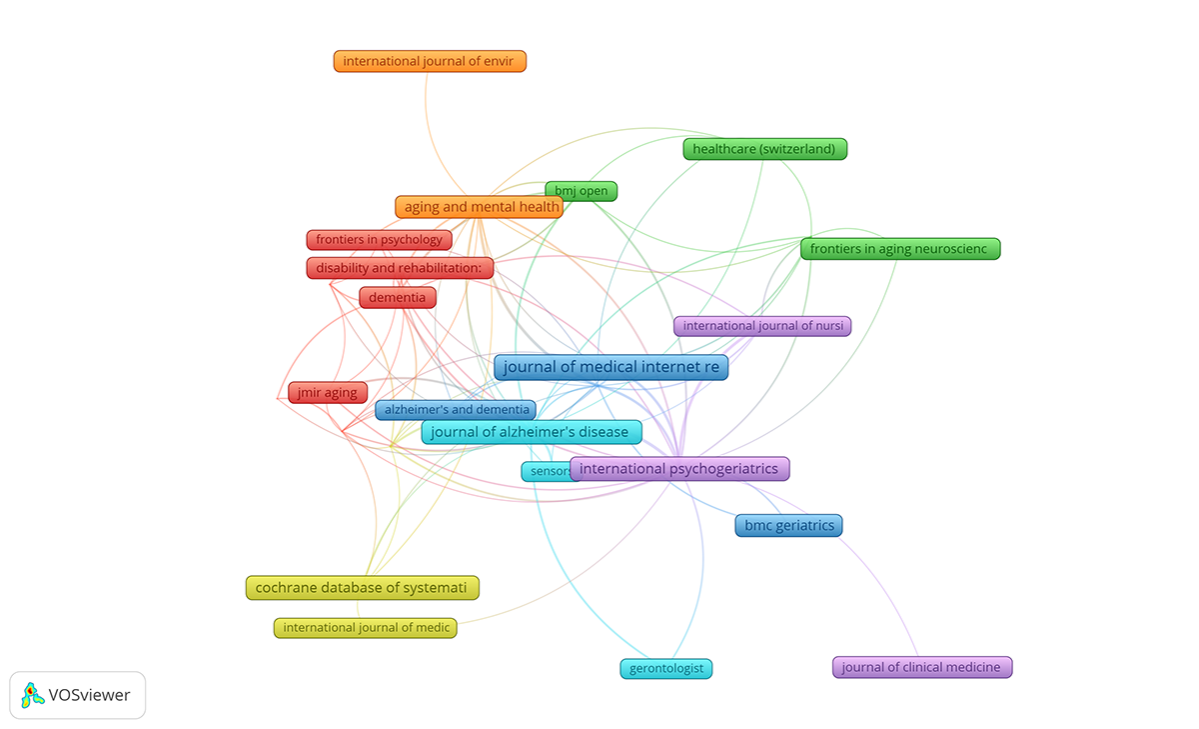
Top contributing and co-cited journals.

Table 1 and Figure 2 present our results on the authors with the most publications and the journals with the highest citations in the field of dementia and DHTs based on our included reviews. The Journal of Medical Internet Research had the highest number of reviews (34/663, 5.12%), with the highest citation count from all journals provided in the ranking (1052). Similarly, the Journal of Alzheimer’s Disease and the Cochrane Database of Systematic Reviews showed substantial metrics regarding published reviews and citation count. Note that the average publication year widely varied across ranked journals. The Ageing Research Reviews (IF_2022=13.1) was the journal with highest IF, while the lowest was the Geriatric Nursing (IF=2.7). Six distinct clusters were identified represented in red, green, blue, yellow, purple, orange, and cyan, each corresponding to a unique collaborative or thematic pattern. A complete analysis of the identified clusters is reported as a legend to Table 1.

As seen in Figure 2, the analysis of clusters revealed that cluster 1 (in red) included journals like *Dementia*, *Disability and Rehabilitation: Assistive Technology*, and *Frontiers in Psychology*, which focus on psychological, social, and rehabilitation features of dementia care, particularly the integration of dementia to assistive technologies. Cluster 2 (in green) contained journals like *Healthcare (Switzerland)* and *Frontiers in Aging Neuroscience*, which were likely to be associated with health care delivery and neuroscience aspects of dementia care, providing a focused and tailored analysis using medical and biological components attributed to DHTs. In cluster 3 (in blue), we observed the presence of highly influential journals like the *Journal of Medical Internet Research* and *International Psychogeriatrics*, whose primary focus relates to internet-based research, psychogeriatrics, and perhaps the broader implications of DHTs in dementia care. Cluster 4 (in yellow) featured journals like the Cochrane Database of Systematic Reviews and *Ageing Research Reviews*, representing evidence-based reviews and systematic approaches to aging and dementia, emphasizing the fundamental role of rigorous evidence synthesis in the development and validation of DHTs in dementia care. In cluster 5 (in purple), journals like *International Journal of Nursing Studies* and *Journal of Clinical Medicine*, were found, which mainly publish research focused on the clinical and nursing aspects of dementia care as well as their integration with DHTs into routine clinical practice. Cluster 6 (in cyan) contained journals like the *Journal of Alzheimer’s Disease* and *Sensors*, with a particular focus on the interface between dementia research and sensor technologies, potentially emphasizing the role of wearable and remote monitoring devices in dementia care. Finally, the small cluster 7 (in orange) represented a distinguished group of journals, highlighting specific thematic focus within the field of dementia care and DHTs.

**Table 1:** Most productive and most cited journals in the field of dementia and digital health technologies.

Journal	Cluster	Links	Total link strength	Documents	Citations	Normalized number of citations	Average citations	Average normalized number of citations
*Journal of Medical Internet Research* (IF^a^=7.4)	3	8	15	34	1052	33.965	30.941	0.999
*Journal of Alzheimer’s Disease* (IF=4.0)	6	18	41	20	743	18.721	37.150	0.936
Cochrane Database of Systematic Reviews (IF=8.4)	4	6	6	18	860	25.357	47.778	1.409
*International Psychogeriatrics* (IF=7.0)	5	18	51	16	936	16.991	58.500	1.062
*International Journal of Geriatric Psychiatry* (IF=4.0)	3	15	31	14	835	12.498	59.643	0.893
*Ageing Research Reviews* (IF=13.1)	4	9	16	13	567	21.866	43.615	1.682
*Aging and Mental Health* (IF=3.4)	7	15	27	13	649	12.201	49.923	0.939
*BMCGeriatrics* (IF=4.1)	3	2	4	13	712	13.862	54.769	1.066
*International Journal of Environmental Research and Public Health* (IF=4.6)	7	1	1	11	104	9.472	9.455	0.861
*Dementia* (IF=4.1)	1	10	22	10	193	10.561	19.300	1.056
*Disability and Rehabilitation: Assistive Technology* (IF=N/A^b^)	1	7	12	10	62	8.924	6.200	0.892
*JMIRAging* (IF=4.9)	1	5	9	10	128	5.860	12.800	0.586
*Journal of the American Medical Directors Association* (IF=7.6)	1	11	13	10	783	11.987	78.300	1.199
*Health care (Switzerland)* (IF=N/A)	2	3	3	9	76	3.286	8.444	0.365
*Journal of Clinical Medicine* (IF=3.9)	5	1	1	9	168	7.497	18.667	0.833
*Frontiers in Aging Neuroscience* (IF=4.8)	2	2	2	8	795	13.445	99.375	1.681
*Geriatric Nursing* (IF=2.7)	2	9	13	8	90	4.099	11.250	0.512
*BMJOpen* (IF=2.9)	2	7	16	7	414	6.144	59.143	0.878
*Gerontologist* (IF=5.7)	6	2	5	7	116	5.492	16.571	0.785
*International Journal of Nursing Studies* (IF=8.1)	5	5	13	7	116	6.658	16.571	0.951
*JMIR Serious Games* (IF=4.0)	1	3	5	7	128	9.206	18.286	1.315
*Clinical Interventions in Aging* (IF=3.6)	1	7	11	6	221	3.767	36.833	0.628
*International Journal of Medical Informatics* (IF=4.9)	4	2	2	6	417	7.725	69.500	1.288
*Alzheimer’s and Dementia* (IF=N/A)	3	6	7	5	590	9.349	118.000	1.870
*Frontiers in Psychology* (IF=3.8)	1	5	6	5	245	7.095	49.000	1.419
*Journal of Clinical Nursing* (IF=4.2)	2	4	4	5	91	2.838	18.200	0.568
*Sensors* (IF=3.9)	6	3	6	5	73	4.463	14.600	0.893

^a^IF: impact factor.

^b^N/A: not applicable.

### Top Contributing Countries and Regions

**Figure 3:**
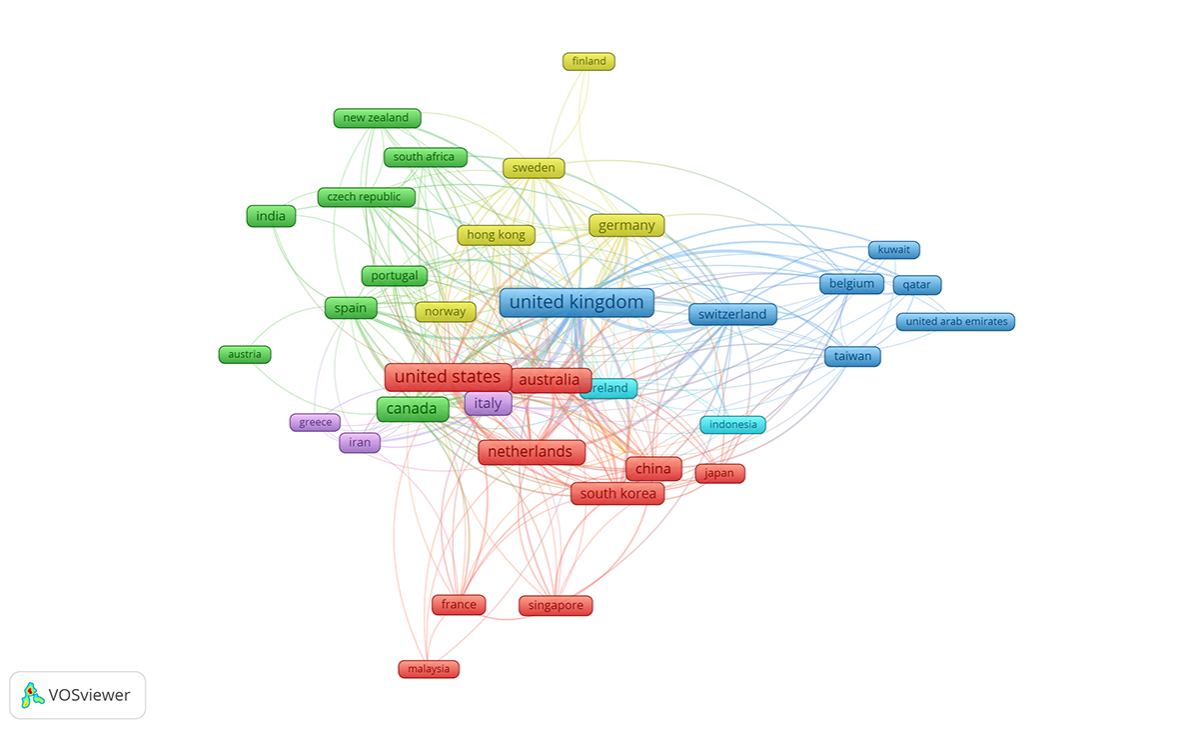
Most prolific countries in the field of dementia and digital health technologies.

Researchers from 66 countries published reviews on the intersection between dementia and DHTs, and Table 2 details the number of citations, documents, and the average number of publications per year, etc. The top 5 countries with evidence-based research in this arena are the United Kingdom (139/663, 20.9%), United States (105/663, 15.8%), Australia (66/663, 9.9%), Canada (61/663, 9.2%), and the Netherlands (53/663, 7.9%). Column 3 of Table 2 shows the country coauthorship networks, particularly evidencing the chord diagram of country cooperation networks ranked by total link strength (Figure 3). The United Kingdom had the highest number of total link strengths, followed by the United States, the Netherlands, Australia, and Switzerland (335, 155, 145, 142, 128, respectively). Countries with the highest number of citations included the United Kingdom (n=6004), United States (n=4911), Australia (n=3491), the Netherlands (n=2647), and Canada (n=1758). Cluster analysis identified 6 different research clusters within the intersection between DHTs and dementia care. Explanation related to identified clusters is available in Table 2.

As seen in Figure 3, cluster 1 (in red) shows that North America and East Asian countries are powerhouses on the research in the interface between DHTs and dementia care, cluster 2 (in green), represents diverse global contributors with a focus on European and South Asian countries, cluster 3 (in blue) suggests leaders in the European and Middle Eastern research hubs, cluster 4 (in yellow) flags Northern European and Nordic countries as research hubs, cluster 5 (in purple) represents Southern Europe and Middle Eastern countries as emerging players in research with growing interest in advancing research in the intersection of DHTs and dementia care, and cluster 6 (in cyan), represents smaller and more focused contributors with specific expertise (in terms of citations).

**Table 2:** Most prolific countries in the field of dementia and digital health technologies.

Country	Cluster	Links	Total link strength	Documents	Citations	Normalized number of citations	Average citations	Average normalized number of citations
United Kingdom	3	31	335	139	6004	150.219	43.194	1.081
United States	1	27	155	105	4911	99.146	46.771	0.944
Australia	1	25	142	66	3491	88.948	52.894	1.348
Canada	2	25	86	61	1758	51.011	28.820	0.836
The Netherlands	1	24	145	53	2647	52.992	49.943	1.000
Italy	5	25	98	49	1709	51.741	34.878	1.056
China	1	21	68	47	797	36.754	16.957	0.782
Germany	4	22	80	38	829	32.881	21.816	0.865
South Korea	1	20	84	28	721	57.516	25.750	2.054
Spain	2	22	59	27	1682	43.560	62.296	1.613
Switzerland	3	28	128	27	1013	23.247	37.519	0.861
India	2	5	7	26	547	64.437	21.039	2.478
Ireland	6	17	25	17	665	17.868	39.118	1.051
Sweden	4	19	42	17	681	21.386	40.059	1.258
Hong Kong	4	11	20	16	337	12.894	21.063	0.806
Norway	4	18	54	16	716	15.065	44.750	0.942
Singapore	1	12	25	16	318	14.130	19.875	0.883
Portugal	2	20	35	15	133	7.120	8.867	0.475
France	1	12	24	14	595	15.008	42.500	1.072
Brazil	7	13	24	13	194	7.003	14.923	0.539
Taiwan	3	16	31	13	263	15.451	20.231	1.189
Belgium	3	18	54	11	701	12.535	63.727	1.140
Iran	5	13	26	11	148	8.837	13.455	0.803
Japan	1	11	24	10	143	5.805	14.300	0.581
New Zealand	2	11	18	8	63	5.008	7.875	0.626
South Africa	2	11	26	8	220	14.498	27.500	1.812
Czech Republic	2	15	36	6	133	2.717	22.167	0.453
Qatar	3	6	20	6	65	7.624	10.833	1.271
Greece	5	6	7	5	139	8.012	27.800	1.602
Indonesia	6	8	10	5	25	2.281	5.000	0.456
Kuwait	3	6	16	5	59	6.930	11.800	1.386
United Arab Emirates	3	4	4	5	65	5.843	13.000	1.169
Austria	2	2	2	4	49	3.914	12.250	0.979
Finland	4	3	3	4	249	5.427	62.250	1.357
Malaysia	1	5	5	4	26	0.728	6.500	0.182

### Top Contributing Authors

**Figure 4:**
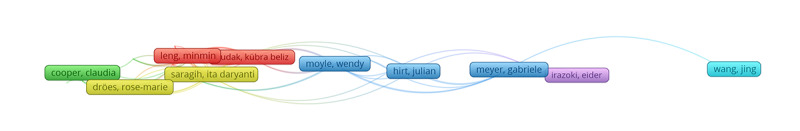
The most prolific authors in the field of dementia and digital health technologies.

Between 2002 and 2024, a total of 3076 authors have published reviews relating to the intersection between dementia and DHTs (Table 3), including authors who have published ≥3 papers. Seven (0.22%) researchers had published 5 documents based on the identified reviews (Rose-Marie Dröes, Julian Hirt, Minmin Leng, Meyer Gabriele, Ita Daryanti Saragih, Jing Wang, and Zhiwen Wang). Column 4 of Table 3 pertains to the author coauthorship networks, particularly related to the density visualization of the number of publications by the author (Figure 4). Some of the 63 (2.04%) authors in the network were not connected to each other, and the largest set of connected authors consisted of 31 (1%) authors. In Figure 4, the redder the color, the higher the number of publications from the determined investigator, and the closer the distance between authors, the greater the collaboration intensity. With regards to the total link strength between identified authors, the most substantial co-occurrence network was performed by Minmin Leng (n=11), Mingyue Hu (n=11), Julian Hirt (n=10), Li Chen (n=9), and Huiru Yin (n=9).

**Table 3:** The most prolific authors in the field of dementia and digital health technologies.

Author	Cluster	Links	Total link strength	Documents	Citations	Normalized number of citations	Average citations	Average normalized number of citations
Dröes, Rose-Marie	4	5	5	5	190	4.161	38.000	0.832
Hirt, Julian	3	7	10	5	81	2.873	16.200	0.575
Leng, Minmin	1	11	11	5	173	4.723	34.600	0.945
Meyer, Gabriele	3	6	8	5	137	2.424	27.400	0.485
Saragih, Ita Daryanti	4	8	8	5	24	2.591	4.800	0.518
Wang, Jing	6	1	1	5	59	2.920	11.800	0.584
Wang, Zhiwen	1	4	4	5	98	3.343	19.600	0.669
Cooper, Claudia	2	2	2	4	455	3.773	113.750	0.943
Hu, Mingyue	1	11	11	4	204	5.287	51.000	1.322
Koh, Wei Qi	1	4	4	4	112	6.307	28.000	1.577
Lee, Bih-O	4	8	8	4	22	2.159	5.500	0.540
Moyle, Wendy	3	7	7	4	170	3.664	42.500	0.916
Robinson, Louise	2	5	5	4	160	2.539	40.000	0.635
Woods, Bob	6	3	3	4	331	14.955	82.750	3.739
Wu, Bei	6	1	1	4	115	2.509	28.750	0.627
Beer, Thomas	3	7	8	3	80	2.657	26.667	0.886
Budak, Kübra Beliz	1	6	6	3	47	4.523	15.667	1.508
Casey, Dympna	2	5	5	3	85	4.741	28.333	1.580
Chen, Li	1	9	9	3	82	2.191	27.333	0.730
Felding, Simone Anna	1	6	6	3	47	4.523	15.667	1.508
Feng, Hui	4	2	2	3	139	4.213	46.333	1.404
Irazoki, Eider	5	2	3	3	107	3.970	35.667	1.323
Jones, Cindy	3	7	7	3	153	2.808	51.000	0.936
Livingston, Gill	2	8	8	3	444	4.968	148.000	1.656
Meyer, Claudia	2	3	3	3	41	2.369	13.667	0.790
Prosperini, Luca	2	1	1	3	125	3.407	41.667	1.136
Roes, Martina	3	2	2	3	33	3.817	11.000	1.272
Toribio-Guzmán, José Miguel	5	2	3	3	107	3.970	35.667	1.323
Van Der Roest, Henriëtte	5	5	5	3	95	4.084	31.667	1.361
Van Straten, Annemieke	4	5	5	3	173	3.111	57.667	1.037
Yin, Huiru	1	9	9	3	82	2.191	27.333	0.730

### Top Contributing Institutions

**Figure 5:**
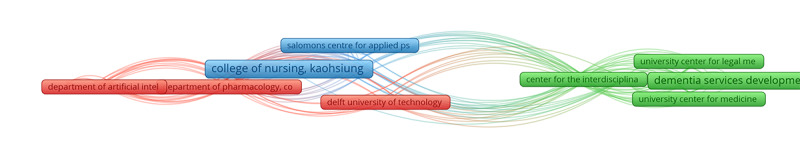
The most prolific organizations in the field of dementia and digital health technologies.

A total of 2218 institutions or academic departments (organizations) have produced reviews on the interface between dementia and DHTs (Table 4). The analysis evidenced that the College of Nursing, Kaohsiung Medical University, Kaohsiung in Taiwan (5/2218, 0.22% documents) and the Dementia Services Development Centre Wales, Bangor University, Bangor in the United Kingdom (4/2218, 0.18% documents) were the most productive centers in the field of digital health and telemedicine. In relation to the highest total number of citations from each organization, 3 (0.13%) institutions from the Netherlands had the highest citation number (n=396). These institutions are Delft University of Technology, Delft Bio-Robotics Lab, in Delft, Maastricht University, Care and Public Health Research Institute, in Maastricht, and Zuyd University of Applied Sciences, Research Centre for Technology in Care, in Heerlen. Column 5 presents the institution coauthorship networks and the chord diagram of the institution cooperation network. In Figure 5, it is worth reiterating that the size of the nodes represents the number of publications per institution. The distance between each node as well as the thickness of the link represents the strength of cooperation between institutions. Cluster analysis is fully described in Table 4. However, the identified clusters represent 1 (in red) institution leading in technological innovation and multidisciplinary research in DHTs; 2 (in green) institutions that focuses on health care implementation, gerontology, and ethics; and 3 (in blue) institutions at the intersection of nursing and applied psychology.

**Table 4:** The most prolific organizations in the field of dementia and digital health technologies.

Organization	Cluster	Links	Total link strength	Documents	Citations	Normalized number of citations	Average citations	Average normalized number of citations
College of Nursing, Kaohsiung Medical University, Kaohsiung, Taiwan	3	11	11	5	24	25.912	4.8	0.5182
Dementia Services Development Centre Wales, Bangor University, Bangor, United Kingdom	2	7	7	4	264	145.713	66	36.428
International Graduate Academy, Institute for Health and Nursing Science, Medical Faculty, Martin Luther University Halle-Wittenberg, Halle (Saale), Germany	2	20	27	2	43	15.234	21.5	0.7617
School of Nursing, Jilin University, Changchun, China	1	13	13	2	68	17.222	34	0.8611
Center for The Interdisciplinary Study of Gerontology and Vulnerability (Cigev), University of Geneva, Geneva, Switzerland	2	16	16	1	24	12.095	24	12.095
Delft University of Technology, Delft Bio-Robotics Lab, Delft, Netherlands	1	10	10	1	396	22.693	396	22.693
Depart of Geriatrics, University of Basel, Switzerland	2	6	7	1	164	20.046	164	20.046
Department of Artificial Intelligence, Korea University, Seoul, South Korea	1	7	7	1	4	0.8643	4	0.8643
Department of Computer Science, Humantech Institute, University of Applied Sciences and Arts Western Switzerland, Fribourg, Switzerland	2	6	7	1	164	20.046	164	20.046
Department of Developmental Psychology, Tilburg University, Tilburg, The Netherlands	2	16	16	1	24	12.095	24	12.095
Department of Health Policy and Management, College of Health Sciences, Korea University, South Korea	1	7	7	1	4	0.8643	4	0.8643
Department of Management, Technology and Economics, Eth Zürich, Zurich, Switzerland	3	12	12	1	0	0	0	0
Department of Pharmacology, College of Basic Medical Sciences, Jilin University, Changchun, China	1	13	13	1	65	10.739	65	10.739
Department of Psychology, University of Geneva, Geneva, Switzerland	2	16	16	1	24	12.095	24	12.095
Division of Health and Medical Sciences, The Cyber University of Korea, Seoul, South Korea	1	7	7	1	4	0.8643	4	0.8643
Fooder Ltd, London, United Kingdom	2	6	7	1	164	20.046	164	20.046
Institute for Biomedical Ethics, Faculty of Medicine, University of Basel, Basel, Switzerland	2	6	7	1	164	20.046	164	20.046
Institute for Health and Society, Medical College of Wisconsin, Milwaukee, WI, United States	2	6	7	1	164	20.046	164	20.046
Institute for Implementation Science in Health Care, University of Zurich, Zurich, Switzerland	3	12	12	1	0	0	0	0
Institute of Biomedical Ethics and History of Medicine, University of Zurich, Zurich, Switzerland	3	12	12	1	0	0	0	0
Institute of Technology Management, University of St Gallen, St Gallen, Switzerland	3	12	12	1	0	0	0	0
Maastricht University, Care and Public Health Research Institute, Maastricht, The Netherlands	1	10	10	1	396	22.693	396	22.693
Salomons Centre For Applied Psychology, Canterbury Christ Church University, Kent, United Kingdom	3	5	5	1	72	0.8801	72	0.8801
School of Mechanical Science and Engineering, Jilin University, Changchun, China	1	13	13	1	65	10.739	65	10.739
School of Nursing and Midwifery, University of Wollongong, Wollongong, Australia	1	4	4	1	176	16.651	176	16.651
School of Nursing, Southern Medical University, Guangzhou, China	1	13	13	1	65	10.739	65	10.739
School of Psychology, University of Wollongong, Wollongong, Australia	1	4	4	1	176	16.651	176	16.651
Swiss National Center of Competences in Research Lives—Overcoming Vulnerability, Life-Course Perspectives, Lausanne and Geneva, Switzerland	2	16	16	1	24	12.095	24	12.095
The First Hospital of Jilin University, Changchun, China	1	13	13	1	65	10.739	65	10.739
Transdisciplinary Major in Learning Health Systems, Department of Public Health Science, Graduate School, Korea University, South Korea	1	7	7	1	4	0.8643	4	0.8643
University Center for Legal Medicine, University of Geneva, Switzerland	2	6	7	1	164	20.046	164	20.046
University Center for Medicine of Aging, Felix Platter Hospital, Basel, Switzerland	2	6	7	1	164	20.046	164	20.046
University of St Gallen, St Gallen, Switzerland	3	12	12	1	0	0	0	0
Zuyd University, Research Centre for Technology in Care, Heerlen, The Netherlands	1	10	10	1	396	22.693	396	22.693

### Analysis of Co-Occurring Keywords

**Figure 6:**
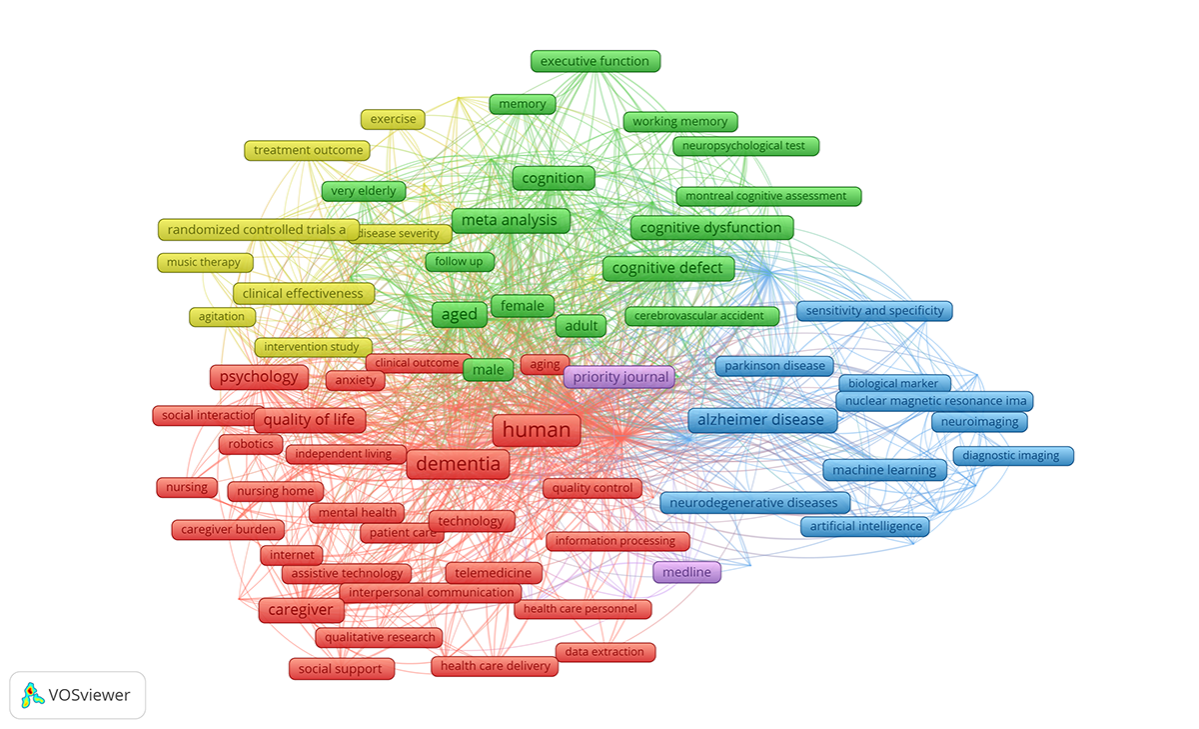
Analysis of co-occurring keywords.

On the basis of the co-occurrence network, a total of 2808 indexed keywords were found with each keyword being used at least 20 times. Table 5 illustrates the keyword co-occurrence network of reviews focusing on dementia and DHTs. The network map contains 5 clusters of 101 items, 4159 links, and 43,365 total link strengths. “Human” was the most frequently occurring keyword (523/2808, 18.63%), followed by “dementia” (385/2808, 13.71%), “systematic review” (327/2808, 11.65%), “review” (327/2808, 11.65%), and “aged” (181/2808, 6.45%). Table 5 presents the top 97 indexed keywords. These keywords are divided into 5 clusters (Figure 6), and its highlighted topographical zones.

**Table 5:** Top 97 most frequently used keywords.

Indexed term	Cluster	Links	Total link strength	Occurrences	Average citations	Average normalized number of citations
Human	1	96	5288	523	38.971	1.0128
Dementia	1	96	3823	385	35.494	0.890
Systematic review	1	96	3938	327	43.810	1.082
Review	1	96	3559	297	41.003	1.060
Aged	2	96	2285	181	39.707	1.014
Cognitive defect	2	96	1997	155	38.032	1.041
Alzheimer disease	3	96	1835	152	39.243	1.190
Quality of life	1	90	1692	145	32.435	0.921
Caregiver	1	81	1359	138	40.290	0.886
Meta-analysis	2	95	1655	133	32.699	1.013
Psychology	1	90	1409	126	45.310	0.885
Cognition	2	95	1620	117	41.615	1.172
Mild cognitive impairment	3	95	1621	113	44.460	1.168
Cognitive dysfunction	2	92	1304	101	33.188	1.059
Depression	1	91	1167	90	46.867	1.137
Female	2	96	1287	87	23.253	0.864
Male	2	96	1224	83	23.169	0.843
Article	3	96	957	75	53.213	1.103
Procedures	4	96	851	74	64.946	1.279
Priority journal	5	95	831	70	62.743	1.000
Adult	2	94	909	65	19.415	0.917
Daily life activity	2	89	846	65	35.662	1.082
Outcome assessment	1	94	832	64	49.344	1.008
Virtual reality	2	91	778	61	29.771	0.933
Clinical effectiveness	4	88	780	56	59.196	1.086
Telemedicine	1	94	642	56	41.143	1.026
Neurodegenerative diseases	3	77	331	53	17.698	1.042
Mini Mental State Examination	2	94	746	48	39.063	1.155
Social support	1	73	542	48	49.938	0.960
Machine learning	3	75	524	44	51.773	1.959
MEDLINE	5	91	603	44	31.068	0.887
Randomized controlled trials as topic	4	78	594	44	54.955	0.979
Executive function	2	70	616	43	53.326	1.369
Technology	1	83	394	43	26.116	0.745
Aging	1	93	545	42	42.857	1.059
Anxiety	1	85	533	42	46.667	1.204
Health care delivery	1	79	392	38	26.290	0.949
Memory	2	80	539	38	48.342	1.185
Middle aged	2	82	539	38	54.868	1.142
Quality control	1	93	558	38	46.474	1.152
Very elderly	2	84	586	37	40.811	1.046
Artificial intelligence	3	65	405	36	24.972	1.400
Deep learning	3	56	406	36	34.417	2.717
Interpersonal communication	1	77	416	36	38.306	1.286
Mental health	1	78	417	36	51.583	1.311
Neuroimaging	3	62	455	36	48.139	1.682
Nuclear magnetic resonance imaging	3	70	465	35	32.457	1.535
Patient care	1	86	390	35	52.229	1.962
Sensitivity and specificity	3	69	454	35	21.914	1.045
Treatment outcome	4	75	456	35	77.029	1.380
Activities of daily living	2	78	439	33	25.849	0.802
Mental disease	1	80	414	33	45.333	1.195
Social interaction	1	71	436	33	40.303	1.188
Cognitive therapy	4	78	438	32	94.688	1.348
Parkinson disease	3	83	422	32	30.344	1.305
Aged ≥80 years	2	79	437	30	65.367	1.024
Caregiver burden	1	69	410	30	44.133	1.127
Diagnostic accuracy	3	69	414	30	57.733	1.518
Physical activity	4	83	394	29	38.448	1.059
Exercise	4	69	359	28	63.964	1.193
Nursing home	1	73	358	28	26.143	0.616
Telehealth	1	73	353	28	39.714	0.736
Internet	1	74	307	27	56.000	1.012
Robotics	1	68	299	27	55.889	1.106
Assistive technology	1	67	274	26	40.423	0.796
Nursing	1	62	292	26	44.615	0.817
Preferred Reporting Items for Systematic Reviews and Meta-Analyses	3	82	344	26	6.115	0.484
Qualitative research	1	73	307	26	22.577	0.689
Working memory	2	77	410	26	41.769	0.918
Agitation	4	64	342	25	61.840	1.093
Cerebrovascular accident	2	73	294	25	31.20	1.048
Controlled study	2	84	409	25	24.60	1.343
Follow-up	2	82	354	25	35.640	1.365
Neuropsychological test	2	67	338	25	64.080	1.223
Practice guideline	5	83	325	25	41.480	1.052
PsycINFO	5	77	380	25	36.560	0.806
Self-help devices	1	49	205	25	57.400	0.956
Clinical outcome	1	85	365	24	25.625	0.872
Data extraction	1	84	310	24	24.542	0.975
Health care personnel	1	80	266	24	14.458	0.655
Independent living	1	74	309	24	42.542	0.994
Cognitive rehabilitation	2	68	332	23	40.304	0.818
Convolutional neural network	3	54	326	23	47.000	2.377
Data base	1	82	303	23	27.044	0.958
Music therapy	4	58	266	23	73.565	0.992
Risk factor	3	76	257	23	36.087	1.170
Diagnostic imaging	3	47	256	22	31.727	1.665
Disease severity	4	75	333	22	55.227	1.430
Early diagnosis	3	64	251	22	36.227	0.973
Embase	5	79	328	22	28.500	0.636
Montreal cognitive assessment	2	77	351	22	18.227	0.989
Support vector machine	3	60	295	22	31.773	1.276
Artificial neural network	3	59	293	21	36.000	1.459
Intervention study	4	71	271	21	51.333	0.793
Biological marker	3	57	239	20	48.000	1.800
Information processing	1	86	282	20	49.550	1.377
Positron emission tomography	3	56	284	20	52.950	1.631

As seen in Figure 6, cluster 1 (in red) represents all technological innovation and multidisciplinary research groups, characterized by groundbreaking institutions at the forefront of technological innovation in DHTs. Cluster 2 (in green) includes institutions that represent health care implementation, gerontology, and ethical considerations, while cluster 3 (in blue) represents evidence groups working on the interface of nursing and applied psychology groups.

### Analysis of Reference Co-Citation Network

**Figure 7:**
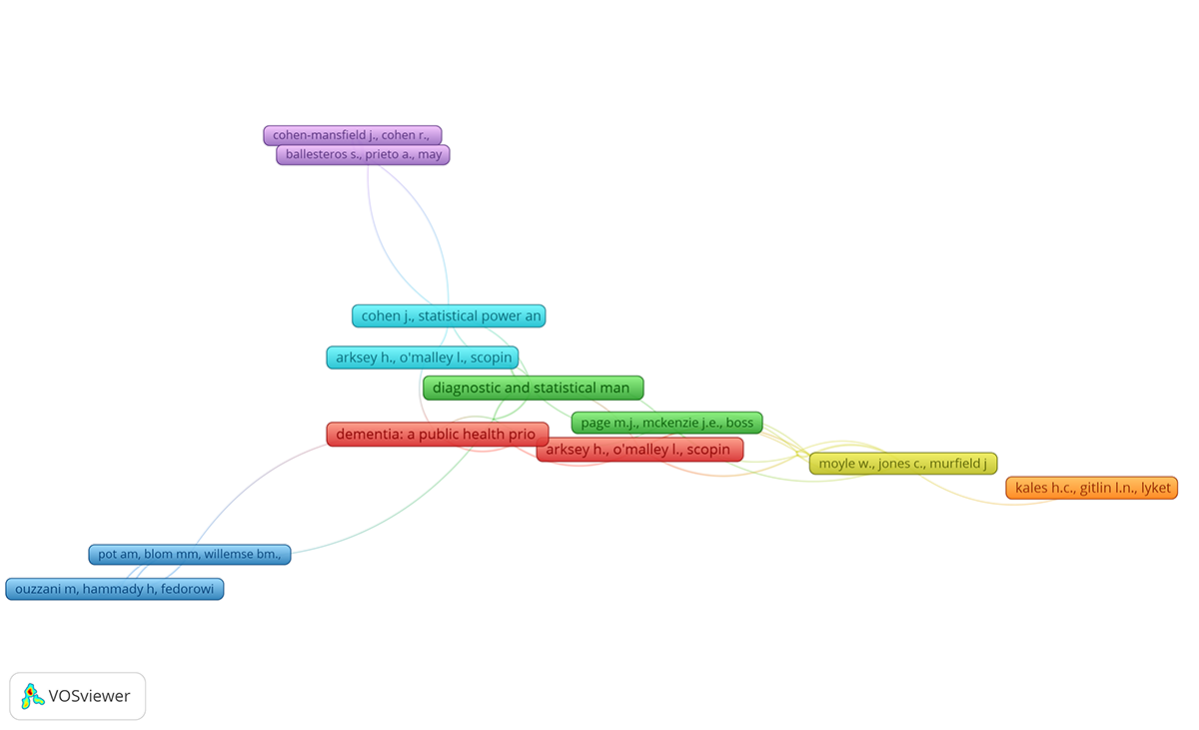
Analysis of cited references (the top 20 most cited references among the included reviews).

In 663 reviews analyzing the interface between dementia and DHTs, 42,758 references were cited **(**Figure 7). On the basis of the statistical assessments performed on VOSviewer to create our co-citation network (parameters: 3 for a minimum number of citations of cited references), 439 (1.02%) cited references were identified. Some of the 439 cited references in the obtained network were not connected. The most extensive set of connected references was 433 (1.01%) (Figure 7). Table 6 presents the top 15 most cited references among the 663 included reviews. The most cited reference was by the WHO, titled “Dementia: a public health priority,” published in 2012 [19].

**Table 6:** Analysis of cited references (the top 15 most cited references among included reviews)a.

Reference locator	Cluster	Links	Total link strength	Citations
1. Dementia: a public health priority [19]	6	35	38	15
2. Arksey H, O’Malley L. Scoping studies: towards a methodological framework [20]	2	64	82	34
3. *Diagnostic and Statistical Manual Of Mental Disorders* [21]	4	51	54	13
4. Arksey H, O'Malley L. Scoping studies: towards a methodological framework [19]	6	32	36	12
5. Kales HC et al. Assessment and management of behavioral and psychological symptoms of dementia [22]	4	25	26	11
6. Cohen J. *Statistical Power Analysis For The Behavioral Sciences* [23]	4	47	50	19
7. Moher D et al. Preferred reporting items for systematic reviews and meta-analyses: the PRISMA statement [24]	3	54	60	20
8. Global action plan on the public health response to dementia 2017-2025 [25]	2	63	104	15
9. Global action plan on the public health response to dementia 2017-2025 [25]	1	32	38	8
10. Arksey H, O’Malley L. Scoping studies: towards a methodological framework [19]	2	35	51	8
11. Bemelmans R et al. How to use robot interventions in intramural psychogeriatric care: a feasibility study [26]	2	38	51	8
12. Lampit A et al. Computerized cognitive training in cognitively healthy older adults: a systematic review and meta-analysis of effect modifiers [27]	3	29	44	8
13. Moher D et al. preferred reporting items for systematic reviews and meta-analyses: the PRISMA statement [24]	2	28	42	7
14. Mordoch E et al. Use of social commitment robots in the care of elderly people with dementia: a literature review [28]	2	20	24	7
15. Moyle W et al. Effect of a robotic seal on the motor activity and sleep patterns of older people with dementia, as measured by wearable technology: a cluster-randomised controlled trial [29]	1	19	21	7
16. Ouzzani M et al. Rayyan-a web and mobile app for systematic reviews [30]	6	35	38	15
17. Bemelmans R et al. How to use robot interventions in intramural psychogeriatric care: a feasibility study [26]	2	64	82	34
18. Abdi J et al. Scoping review on the use of socially assistive robot technology in elderly care [31]	4	51	54	13
19. Astell AJ et al. Technology and dementia: the future is now, dementia and geriatric cognitive disorders [32]	6	32	36	12
20. Page MJ et al. The PRISMA 2020 statement: an updated guideline for reporting systematic reviews [33]	4	25	26	11

^a^Of note, we observed the existence of duplicated references not primarily identified through the software use for performing our bibliometric analysis (VOSviewer). Thus, we consolidated the reported metrics accordingly whenever needed (links, total link strength, and citations) into a single entry to ensure the most accurate representation within the cocitation network assessment.

## Discussion

### Principal Findings

#### Overview

This primary review describes a high-level bibliometric insight into the literature on the existing DHTs and dementia care between 2002 and 2024. An increasing number of reviews (either systematic or scoping) have been published in medical- and technology-related scientific journals (704/704, 100% records). The included reviews suggest that the number of publications covering the intersection of digital health and dementia has increased steadily over time (compound annual growth rate of 26%), with some fluctuations. However, our observations highlight how several research initiatives released in indexed journals have covered similar modalities of digital technologies and research questions. Our findings align with trends reported in several studies, showing that the proliferation of reviews on a single topic leads to duplicated efforts and underscores the need for greater accountability to reduce research waste [34]. In addition, our data highlight the concentration of research originating from high-income settings, presenting an opportunity for broader reflection on the implications of population aging within these countries.

#### Implications for Dementia Care

The challenges associated with the care of people living with dementia, as well as their caregivers, highlight the need for policy makers to reinforce adequate interventions in dementia care delivery. This review serves as a primary, high-level resource for policy makers and academics to enable them to understand and address the growing challenges in dementia care. As reported in the identified overviews of specific modalities of DHTs, multiple digital interventions pose as relevant tools for improving the health-related outcomes of people living with dementia and their caregivers. For instance, multidomain online lifestyle programs have been reported to not only positively affect population brain health outcomes but also potentially contribute to the prevention of dementia (based on findings from the Lifestyle Enrichment for Alzheimer Prevention program) [35]. One overview investigating the role of eye-tracking technologies in evaluating eye movements and pupillometry parameters during different stages of dementia (using machine learning algorithms) suggested the potential for early diagnosis and the monitoring of cognitive decline, in addition to predicting the risk of developing dementia long-term [36]. Likewise, wearable technologies and sensors (including environmental sensor-based systems and video systems) are suggested to reduce falls and fall risk for people living with dementia or mild cognitive impairment [37].

#### Limitations in Current Research Approaches

Findings from this bibliometric review have ascertained the high number of reviews evaluating the impact of DHTs on dementia care. However, we have considered that there are limitations pertaining to the evidence-making process, which might not prove adequate. We found several reviews covering similar research questions (such as reviews evaluating the use of exergaming or AI in dementia care), exploring equivalent methodological approaches (such as similar research questions and eligible populations), and even underlying parallel outcomes of interest. For instance, 3 systematic reviews published in 2017, 2020, and 2023 investigated the impact of exergames on individuals with cognitive impairment to minimize the thematic level [38-40]. While the selected cases held slightly different foci, the core elements proved virtually identical, particularly the results, interpretations, and future directions for research stakeholders. This finding echoes the concerns stressed by multiple evidence producers, highlighting the potential for research redundancy in the reported results [41-44]. We recommend several strategies to address this concern, emphasizing that future research on digital health in dementia care should build on existing evidence and explore complementary knowledge gaps. In addition, we advocate for the use of standardized guidelines for conducting systematic reviews, such as those in the Cochrane Handbook of Systematic Reviews and other authority sources in the field [45]. Avoiding research duplication can be facilitated by creating a centralized research database that serves as a repository for ongoing and complemented initiatives. This would help investigators worldwide to identify knowledge gaps and prevent redundant efforts [46,47]. Moreover, fostering research collaboration and communication through interdisciplinary partnerships and international online forums can further reduce duplication [46,47]. Preregistration of studies and the adoption of open science practices enhance transparency and allow researchers to identify existing projects.

We also highlight the critical role of scientific diplomats in fostering international collaboration and multilateral partnerships. By using concepts, norms, and values in scientific diplomacy, typically used in political diplomacy, we take actions to facilitate international scientific collaboration (eg, facilitating the negotiation of research and development agreements and exchange programs or services, or enabling the establishment of international research infrastructure) [48]. Although not frequently known by senior researchers, this labor class has becoming more frequently needed and common over the last years as scientific diplomats are a critical part of fostering and initiating discussions where science, innovation, and technological advancement intersect with international relations and policy [49]. These professionals are commonly active researchers who use diplomatic responsibilities to not only influence (locally or internationally), but to also represent national interests (commonly known as diplomat scientist) to other nations, or they might potentially become specialized in a particular domain of expertise (ie, science, technology, and innovation policies in international collaboration) [50]. In addition, scientific diplomats help establish shared and standardized protocols, promote the adoption of best practices, and ensure the long-term sustainability of international research network [51]. These efforts contribute to more efficient and impactful scientific strategies on a global scale.

#### Cluster Analysis and Research Trends

We selected specific clusters for detailed and comprehensive analysis. Notably, our analysis of these clusters reveals relevant and timely discussions, particularly regarding their alignment with current trends in DHTs for dementia care and their implications for future research directions.

First, regarding the clusters identified in our assessment of the most cited journals in the field of dementia and DHTs, we observed a diverse landscape. Interdisciplinary collaboration, evidence-based development, and personalized care emerged as major themes. Notably, the clusters highlight the integration of psychological, social, clinical, and technological aspects, with increasing emphasis on using DHTs for mental health support (for both patients or formal and informal health care providers), sensor technologies for real-time monitoring, and systematic reviews to evaluate the efficacy of these tools.

On the basis of these findings, future research should prioritize not only collaborative research development but also the synergic integration of DHTs into health care systems, particularly in nursing and clinical practice, while always considering ethical concerns, such as privacy and the specific implementation challenges for different labor sectors. Another cluster we analyzed involved the most prolific countries in the field of dementia and DHTs. This revealed a complex global network, with leading research hubs forming distinct clusters. High-income countries, such as the United Kingdom (cluster 3), and the United States and Australia (cluster 1), stand out with the highest number of reviews and citations, underscoring their central role in advancing scientific knowledge related to DHTs for dementia care. Emerging middle- or high-income countries, including India and South Korea, respectively, are becoming notable research hubs in Asia, expanding DHT-related research beyond traditional Western centers.

Our data also point to growing international collaboration, particular among European countries, likely driven by concerns about populational aging in the region and the European Union funding programs that require a multicenter approach to research. This global distribution emphasizes the importance of fostering international research partnerships to ensure that DHT innovations are accessible and applicable worldwide, not just in high-income countries.

Finally, our cluster analysis highlighted key institutions and research groups active in the field of dementia and DHTs. These findings underscore the importance of a multidisciplinary approach to advancing dementia care through digital health, stressing the need for collaboration across technology, health care services, ethics, and human factors to design effective, sustainable solutions.

This review predominantly included studies conducted and produced by researchers located in high-income countries. The impact of dementia and neurocognitive impairment has been noteworthy in nations where the gross national income per capita is more than US $12,376 [52]. However, the rising prevalence of dementia is not limited to high-income settings. Recent estimates suggest that by 2030, more than 75 million individuals with dementia will reside in low- and middle-income countries [53]. While our analysis included studies from various nations, there remains a need for more global and collaborative effort beyond the regions currently leading this research. These efforts should focus on evaluating the diverse roles of DHTs in dementia care, taking into action regional specificities, needs, and expectations. Furthermore, this review highlights global regions that do not feature highly in the results; this is a call for action to researchers, organizations, and policy makers in the respective regions (Latin America, Asia, Africa, Central and Eastern Europe, and so forth) to explore and conduct research in this arena.

The coauthorship and collaboration network analysis highlighted the most prolific collaboration between authors and institutions with the most relevant reviews on DHTs for dementia care. Rose-Marie Dröes, Julian Hirt, and Minmin Leng emerged as critical global investigators. At the same time, institutional productivity showed that the College of Nursing, Kaohsiung Medical University, Kaohsiung, Taiwan, and the Dementia Services Development Centre Wales, Bangor University, United Kingdom are leading contributors in this arena. By understanding this trend in productivity over time, we can further evaluate their influence on this inter and multidisciplinary field.

#### Implications for Future Research, Clinicians, Researchers, and Policy Makers

Our study systematically evaluates the extent to which DHTs have been described as potential interventions in dementia care. For several DHT modalities, particularly those with substantial scientific output, these technologies have provided clinicians with a strong evidence base to inform their clinical practices, enabling them to integrate (or reject) these tools for people living with dementia [54]. However, further detailed assessments are needed to determine whether various digital interventions adequately address the physical, cognitive, and social needs of people living with dementia and their caregivers.

Our bibliometric analysis highlights the growing and evolving body of literature on the critical role of DHTs in advancing dementia care, particularly within the context of international health priorities, such as those endorsed by the WHO’s Global Digital Health Strategy 2020 to 2025 [55]. Given the rising global incidence of dementia, there is an imperative demand for innovative solutions that can be scaled globally, especially in low- and middle-income countries, where the burden of dementia-related disorders is expected to significantly increase by 2030.

The WHO’s strategy on digital health emphasizes the importance of leveraging DHTs through equitable process, aiming to achieve universal health coverage, enhance research, and improve the quality of care [55]. These principles align directly with the potential of DHTs for dementia care, as observed in our study. With most research initiatives concentrated in high-income countries, future research should focus on the tailored development, long-term evaluation, and implementation of DHTs in diverse settings, including resource-limited environments.

Policy makers must consider the implications of our research for establishing and sustaining international research collaborations, as well as the need for interventions specifically designed to address the challenges faced by different populations. Moreover, the “publish or perish” paradigm prevalent in academia contributes to research duplication, which should be mitigated through various strategies, as previously discussed [56]. These proposed actions are aligned with the WHO’s initiatives for improving global research and advancing industrial technologies, under the Sustainable Development Goals 9. By aligning future research with the priorities outlined in the WHO Global Digital Health Strategy, stakeholders can support the development of comprehensive, evidence-informed public health policies that promote the equitable adoption of DHTs in dementia care.

#### Strengths and Limitations

This bibliometric review has provided evidence of the modalities of DHTs in the context of dementia. The findings highlight domains that still require further evaluation and geographic regions that are leading in this arena but also require further investigation at a local level. In addition, we did not observe any previous systematic reviews evaluating the role of telemedicine and remote telemonitoring interventions on dementia care. There is a paucity in the current body of literature pertaining to the appraisal of the applicability of big data analytics regarding health-related decision-making. These analytics have been shown to provide important clinical insights that have the potential to support the development of effective therapeutic strategies and preventive measures. There is a planned body of work relating to a series of papers whereby the authors intend to evaluate the potential of these 2 modalities of digital interventions, encompassing multiple prospective domains and influencing health, economic, or social outcomes. We believe that by identifying the knowledge gap, we will facilitate the provision of care for both people living with dementia and their caregivers. Moreover, we anticipate future results will alleviate the burden of dementia on international health care systems and optimize resource allocation toward programs that adequately supply patients and social actors involved in care. This in turn has the potential to furnish national governments, local authorities, and municipalities with greater insights to tackle respective priorities and strategies. This too may aid policy makers in identifying financial resources [57] at a local level in the context of age-friendly cities and communities [58].

This review used a solid and comprehensive methodology, although there are limitations. First, in this bibliometric analysis, reviews were included if they were located in the Scopus database, and a few identified records were not indexed in this database. Because we solely focused on the Scopus database, we may have missed key literature that might hinder the generalizability of our findings. However, we believe that the core reported findings would not be significantly changed. Second, while the observed keywords and identifiers analysis offered important insights regarding research topics that have received scholarly attention over time, further detailed assessments of the shortlisted publications may provide more specific information associated with the interface between digital health and dementia care. Third, our study’s selection criterion (exclusively focusing on systematic and scoping reviews) may have slightly introduced selection bias due to the exclusion of narrative and integrative reviews. Although commonly labeled as less rigorous in their methodology, they could still offer some additional perspectives and insights into emerging tendencies, particularly in a field such as digital health, which is constantly evolving. However, we believe that maintaining our work based on better methodologically designed records increases the validity and credibility of our study. In addition, potential publication bias must be acknowledged, especially, with the repeated similar reviews that may overrepresent certain technologies or outcomes. Finally, we did not assess the methodological quality of systematic reviews identified in our screening because of the high volume of eligible reviews. However, we will prioritize the quality appraisal in the process of evidence synthesis by following the PRISMA (Preferred Reporting Items for Systematic reviews and Meta-Analyses) guidelines to report the upcoming systematic review.

### Conclusions

This review indicates that there has been an increase in both dementia- and DHT-related publications since the first review in 2002, with a more prominent growth observed after 2017. Despite the diverse approach of DHT modalities for dementia care, authors have focused on mobile health, virtual reality, AI, and sensor technologies. Analysis revealed prominent authors and institutions studying the intersection between digital health and dementia. High-income countries were identified as the leaders in publishing and maintaining research collaborations on this topic. Overall, this analysis provides an initial point of reference for policy makers and academics who wish to conduct research or collaborate on funding calls to understand the contemporary landscape in a bid to undertake innovative and applied research that will primarily benefit people living with dementia, their caregivers, and service provision and resources. We particularly recommend prioritizing the exploration of underrepresented DHTs for research questions and expanding research in low- and middle-income countries to ensure alignment with global equity principles. In addition, it is essential for the scientific and governmental communities to develop frameworks that effectively integrate DHTs into dementia care, guided by the principles of accessibility, affordability, and user friendliness. This review has illustrated emerging global trends and identified areas where future research should focus, especially considering the current emphasis on high-income countries and the remaining knowledge gaps.

## Appendix

Multimedia Appendix 1 BIBLIO checklist for reporting bibliometric reviews of biomedical literature.

Multimedia Appendix 2 Search strategies. The search was conducted on February 20, 2024.

Multimedia Appendix 3 Identified overviews or umbrella reviews during the second screening phase.

Multimedia Appendix 4 Reasons for excluding shortlisted studies.

Multimedia Appendix 5 References without a digital object identifier.

Multimedia Appendix 6 References not indexed in Scopus.

## Abbreviations

AI: artificial intelligence
DHT: digital health technology
DOI: digital object identifier
IF: impact factor
PRISMA: Preferred Reporting Items for Systematic reviews and Meta-Analyses
WHO: World Health Organization

## Data Availability

The datasets generated and analyzed during this study are available from the corresponding author on reasonable request.
