# Supplementary material for: Use of Digital Health Technologies for Dementia Care: Bibliometric Analysis and Report
Source: JMIR Ment Health. 2025 Feb 10;12:e64445. doi: 10.2196/64445 (PMC11851039; doi:10.2196/64445)
Supplement: Multimedia Appendix 2 [file mental_v12i1e64445_app2.docx]

**Multimedia Appendix 2 – Search strategies - The search was conducted on 20 February 2024**

Search details for Comprehensive Overview of Digital Health Interventions for Dementia: A Synthesis of Systematic and Scoping Reviews Across Prevention, Diagnosis, Treatment, and Care Support Team

By: Lasse Østengaard - Bibliographic databases: Embase (Ovid), Medline (Ovid), Cochrane Database of Systematic Reviews (Cochrane Library), Scopus and Epistemonikos.

**Embase**

| **Embase Classic+Embase <1947 to 2024 February 19>** | | |
| --- | --- | --- |
|  |  |  |
| 1 | exp dementia/ | 464683 |
| 2 | exp delirium/ | 45480 |
| 3 | dement*.mp. | 271791 |
| 4 | deliri*.mp. | 51928 |
| 5 | alzheimer*.mp. | 315313 |
| 6 | (lewy* adj2 bod*).mp. | 23785 |
| 7 | (creutzfeldt or cjd).mp. | 14767 |
| 8 | huntington*.mp. | 39820 |
| 9 | major neurocognitive disorder*.mp. | 546 |
| 10 | binswanger*.mp. | 1145 |
| 11 | korsako*.mp. | 3473 |
| 12 | (pick* adj2 disease).mp. | 8518 |
| 13 | (cerebr* adj2 deteriorat*).mp. | 436 |
| 14 | (frontotemporal adj2 degenerat*).mp. | 5724 |
| 15 | 1 or 2 or 3 or 4 or 5 or 6 or 7 or 8 or 9 or 10 or 11 or 12 or 13 or 14 | 608030 |
| 16 | exp telehealth/ | 92381 |
| 17 | (telemedicine or telehealth or telemonitoring or remote monitoring).mp. | 85978 |
| 18 | (mhealth or m-health or ehealth or e-health or emental or e-mental or e-prescri*).mp. | 23727 |
| 19 | ((Medical or health) adj3 monitor*).mp. | 26142 |
| 20 | exp medical informatics/ | 23893 |
| 21 | ((Medical or biomedical or health or clinical) adj3 informatics).mp. | 30096 |
| 22 | exp technology/ | 279270 |
| 23 | (technolog* or digital*).mp. | 1313204 |
| 24 | exp internet/ | 132477 |
| 25 | computer assisted therapy/ or personal digital assistant/ | 6715 |
| 26 | ((internet or online or web* or tele* or computer or cyberspace or cyber space or remote or virtual or electronic) adj3 (consult* or based or application* or intervention* or program* or therap* or rehabilitat* or prescri*)).mp. | 419435 |
| 27 | exp mobile phone/ or exp mobile application/ | 68481 |
| 28 | (app or apps).mp. | 64557 |
| 29 | (mobile* or portable or phone* or telephone* or smartphone* or cellphone* or smartwatch* or screen or touchscreen or multimedia or multi media).mp. | 720663 |
| 30 | (Gaming or game or exergam*).mp. | 53854 |
| 31 | robot*.mp. | 128202 |
| 32 | (Connected Devices or Smart Devices or Digital Assistant).mp. | 3950 |
| 33 | electronic medical record system/ or electronic medical record/ or exp hospital information system/ | 114434 |
| 34 | ((Health or medical or hospital) adj3 (record* or informati* or data or science)).mp. | 958545 |
| 35 | ((Clinical or medical) adj3 (alert or reminder)).mp. | 1812 |
| 36 | (personalized medicine or personalised medicine or interoperability or decision support).mp. | 139161 |
| 37 | exp social media/ | 52761 |
| 38 | (social media or social network* or Facebook or twitter or youtube or Instagram or flickr or Linkedin or blog* or on-line communit* or online communit* or wiki*).mp. | 106586 |
| 39 | (surveillance or GPS or track* or position* or sensor* or big data or open data or cloud or 4G* or 5G* or bluetooth or wearable* or wireless).mp. | 2382949 |
| 40 | (home adj6 (automat* or monitor*)).mp. | 18043 |
| 41 | exp artificial intelligence/ | 95956 |
| 42 | ((Artificial or machine or deep or hierarchical or ambient or comput* or assist*) adj3 (intelligence or learning)).mp. | 264711 |
| 43 | (Computer adj3 (vision or knowledge)).mp. | 11288 |
| 44 | (Knowledge adj3 (acquisition or representation*)).mp. | 7673 |
| 45 | natural language processing.mp. | 14296 |
| 46 | (AI or NLP).mp. | 79604 |
| 47 | ((Computer or automated) adj3 reasoning).mp. | 249 |
| 48 | 16 or 17 or 18 or 19 or 20 or 21 or 22 or 23 or 24 or 25 or 26 or 27 or 28 or 29 or 30 or 31 or 32 or 33 or 34 or 35 or 36 or 37 or 38 or 39 or 40 or 41 or 42 or 43 or 44 or 45 or 46 or 47 | 5870583 |
| 49 | exp "systematic review"/ or exp meta analysis/ | 583407 |
| 50 | exp "systematic review (topic)"/ or exp "meta analysis (topic)"/ | 75008 |
| 51 | ((systematic or scoping) adj3 (review* or overview*)).ti,ab. | 425079 |
| 52 | (meta analy* or metaanaly* or meta-analy*).ti,ab. | 373615 |
| 53 | 49 or 50 or 51 or 52 | 787543 |
| 54 | 15 and 48 and 53 | 3576 |

**MEDLINE^®^**

| Ovid MEDLINE(R) ALL <1946 to February 19, 2024> | | |
| --- | --- | --- |
|  |  |  |
| 1 | exp Dementia/ | 212406 |
| 2 | exp Delirium/ | 13216 |
| 3 | dement*.mp. | 171747 |
| 4 | deliri*.mp. | 25656 |
| 5 | alzheimer*.mp. | 211169 |
| 6 | (lewy* adj2 bod*).mp. | 13128 |
| 7 | (creutzfeldt or cjd).mp. | 9368 |
| 8 | huntington*.mp. | 23245 |
| 9 | major neurocognitive disorder*.mp. | 359 |
| 10 | binswanger*.mp. | 609 |
| 11 | korsako*.mp. | 1872 |
| 12 | (pick* adj2 disease).mp. | 4569 |
| 13 | (cerebr* adj2 deteriorat*).mp. | 268 |
| 14 | (frontotemporal adj2 degenerat*).mp. | 3960 |
| 15 | 1 or 2 or 3 or 4 or 5 or 6 or 7 or 8 or 9 or 10 or 11 or 12 or 13 or 14 | 373909 |
| 16 | exp Telemedicine/ | 46711 |
| 17 | (telemedicine or telehealth or telemonitoring or remote monitoring).mp. | 61478 |
| 18 | (mhealth or m-health or ehealth or e-health or emental or e-mental or e-prescri*).mp. | 22402 |
| 19 | ((Medical or health) adj3 monitor*).mp. | 21804 |
| 20 | exp Medical Informatics/ | 504353 |
| 21 | ((Medical or biomedical or health or clinical) adj3 informatics).mp. | 23847 |
| 22 | exp Technology/ | 505857 |
| 23 | (technolog* or digital*).mp. | 976394 |
| 24 | exp Internet/ | 99699 |
| 25 | exp Computers, Handheld/ or exp Therapy, Computer-Assisted/ | 60062 |
| 26 | ((internet or online or web* or tele* or computer or cyberspace or cyber space or remote or virtual or electronic) adj3 (consult* or based or application* or intervention* or program* or therap* or rehabilitat* or prescri*)).mp. | 197464 |
| 27 | exp Cell Phone/ or Mobile Applications/ | 31484 |
| 28 | (app or apps).mp. | 47937 |
| 29 | (mobile* or portable or phone* or telephone* or smartphone* or cellphone* or smartwatch* or screen or touchscreen or multimedia or multi media).mp. | 502368 |
| 30 | (Gaming or game or exergam*).mp. | 42889 |
| 31 | robot*.mp. | 82661 |
| 32 | (Connected Devices or Smart Devices or Digital Assistant).mp. | 2172 |
| 33 | exp Medical Records Systems, Computerized/ or exp hospital information system/ | 75418 |
| 34 | ((Health or medical or hospital) adj3 (record* or informati* or data or science)).mp. | 497187 |
| 35 | ((Clinical or medical) adj3 (alert or reminder)).mp. | 1136 |
| 36 | (personalized medicine or personalised medicine or interoperability or decision support).mp. | 75088 |
| 37 | exp Social Media/ | 16931 |
| 38 | (social media or social network* or Facebook or twitter or youtube or Instagram or flickr or Linkedin or blog* or on-line communit* or online communit* or wiki*).mp. | 73158 |
| 39 | (surveillance or GPS or track* or position* or sensor* or big data or open data or cloud or 4G* or 5G* or bluetooth or wearable* or wireless).mp. | 1806531 |
| 40 | (home adj6 (automat* or monitor*)).mp. | 9609 |
| 41 | exp Artificial Intelligence/ | 190243 |
| 42 | ((Artificial or machine or deep or hierarchical or ambient or comput* or assist*) adj3 (intelligence or learning)).mp. | 213802 |
| 43 | (Computer adj3 (vision or knowledge)).mp. | 9787 |
| 44 | (Knowledge adj3 (acquisition or representation*)).mp. | 6331 |
| 45 | natural language processing.mp. | 12132 |
| 46 | (AI or NLP).mp. | 59165 |
| 47 | ((Computer or automated) adj3 reasoning).mp. | 211 |
| 48 | 16 or 17 or 18 or 19 or 20 or 21 or 22 or 23 or 24 or 25 or 26 or 27 or 28 or 29 or 30 or 31 or 32 or 33 or 34 or 35 or 36 or 37 or 38 or 39 or 40 or 41 or 42 or 43 or 44 or 45 or 46 or 47 | 4640018 |
| 49 | exp "systematic review"/ or exp meta-analysis/ | 338887 |
| 50 | exp Systematic Reviews as Topic/ or exp Meta-Analysis as Topic/ | 37194 |
| 51 | ((systematic or scoping) adj3 (review* or overview*)).ti,ab. | 355144 |
| 52 | (meta analy* or metaanaly* or meta-analy*).ti,ab. | 296214 |
| 53 | 49 or 50 or 51 or 52 | 526807 |
| 54 | 15 and 48 and 53 | 2017 |

**Cochrane Library**

|  |  |  |
| --- | --- | --- |
| ID | Search | Hits |
| #1 | MeSH descriptor: [Dementia] explode all trees | 9235 |
| #2 | MeSH descriptor: [Delirium] explode all trees | 1533 |
| #3 | (dement*):ti,ab,kw | 17753 |
| #4 | (deliri*):ti,ab,kw | 5745 |
| #5 | (alzheimer*):ti,ab,kw | 14169 |
| #6 | (lewy* NEAR/1 bod*):ti,ab,kw | 538 |
| #7 | (creutzfeldt or cjd):ti,ab,kw | 69 |
| #8 | (huntington*):ti,ab,kw | 815 |
| #9 | (major neurocognitive disorder*):ti,ab,kw | 761 |
| #10 | (binswanger*):ti,ab,kw | 9 |
| #11 | (korsako*):ti,ab,kw | 79 |
| #12 | (pick* NEAR/1 disease):ti,ab,kw | 129 |
| #13 | (cerebr* NEAR/1 deteriorat*):ti,ab,kw | 11 |
| #14 | (frontotemporal NEAR/1 degenerat*):ti,ab,kw | 18 |
| #15 | #1 or #2 or #3 or #4 or #5 or #6 or #7 or #8 or #9 or #10 or #11 or #12 or #13 or #14 | 32251 |
| #16 | MeSH descriptor: [Telemedicine] explode all trees | 4744 |
| #17 | (telemedicine or telehealth or telemonitoring or "remote monitoring"):ti,ab,kw | 10681 |
| #18 | (mhealth or m-health or ehealth or e-health or emental or e-mental or e-prescri*):ti,ab,kw | 4400 |
| #19 | ((Medical or health) NEAR/2 monitor*):ti,ab,kw | 1695 |
| #20 | MeSH descriptor: [Medical Informatics] explode all trees | 14699 |
| #21 | ((Medical or biomedical or health or clinical) NEAR/2 informatics):ti,ab,kw | 458 |
| #22 | MeSH descriptor: [Technology] explode all trees | 9050 |
| #23 | (technolog* or digital*):ti,ab,kw | 51579 |
| #24 | MeSH descriptor: [Internet] explode all trees | 6471 |
| #25 | MeSH descriptor: [Computers, Handheld] explode all trees | 1525 |
| #26 | MeSH descriptor: [Therapy, Computer-Assisted] explode all trees | 3190 |
| #27 | ((internet or online or web* or tele* or computer or cyberspace or "cyber space" or remote or virtual or electronic) NEAR/2 (consult* or based or application* or intervention* or program* or therap* or rehabilitat* or prescri*)):ti,ab,kw | 40253 |
| #28 | MeSH descriptor: [Cell Phone] explode all trees | 3421 |
| #29 | MeSH descriptor: [Mobile Applications] this term only | 1898 |
| #30 | (app or apps):ti,ab,kw | 10674 |
| #31 | (mobile* or portable or phone* or telephone* or smartphone* or cellphone* or smartwatch* or screen or touchscreen or multimedia or "multi media"):ti,ab,kw | 71986 |
| #32 | (gaming or game or exergam*):ti,ab,kw | 6579 |
| #33 | (robot*):ti,ab,kw | 7450 |
| #34 | ("Connected Devices" or "Smart Devices" or "Digital Assistant"):ti,ab,kw | 251 |
| #35 | MeSH descriptor: [Medical Records Systems, Computerized] explode all trees | 1174 |
| #36 | MeSH descriptor: [Hospital Information Systems] explode all trees | 863 |
| #37 | ((Health or medical or hospital) NEAR/2 (record* or informati* or data or science)):ti,ab,kw | 31013 |
| #38 | ((Clinical or medical) NEAR/2 (alert or reminder)):ti,ab,kw | 97 |
| #39 | ("personalized medicine" or "personalised medicine" or interoperability or "decision support"):ti,ab,kw | 6263 |
| #40 | MeSH descriptor: [Social Media] explode all trees | 562 |
| #41 | ("social media" or "social network" or "social networks" or Facebook or twitter or youtube or Instagram or flickr or Linkedin or blog* or "on-line community" or "on-line communities" or "online community" or "online communities" or wiki*):ti,ab,kw | 5994 |
| #42 | (surveillance or GPS or track* or position* or sensor* or "big data" or "open data" or cloud or 4G* or 5G* or bluetooth or wearable* or wireless):ti,ab,kw | 104924 |
| #43 | (home NEAR/5 (automat* or monitor*)):ti,ab,kw | 2799 |
| #44 | MeSH descriptor: [Artificial Intelligence] explode all trees | 3108 |
| #45 | ((Artificial or machine or deep or hierarchical or ambient or comput* or assist*) NEAR/2 (intelligence or learning)):ti,ab,kw | 5432 |
| #46 | (Computer NEAR/2 (vision or knowledge)):ti,ab,kw | 211 |
| #47 | (Knowledge NEAR/2 (acquisition or representation*)):ti,ab,kw | 502 |
| #48 | ("natural language processing"):ti,ab,kw | 256 |
| #49 | (AI or NLP):ti,ab,kw | 5911 |
| #50 | ((Computer or automated) NEAR/2 reasoning):ti,ab,kw | 7 |
| #51 | #16 or #17 or #18 or #19 or #20 or #21 or #22 or #23 or #24 or #25 or #26 or #27 or #28 or #29 or #30 or #31 or #32 or #33 or #34 or #35 or #36 or #37 or #38 or #39 or #40 or #41 or #42 or #43 or #44 or #45 or #46 or #47 or #48 or #49 or #50 | 290278 |
| #52 | #15 and #51 | 5795 |
| #53 | #52 in Cochrane Reviews | 74 |

**Scopus^®^**

| 1 | ( ( TITLE-ABS-KEY ( ( dement* OR deliri* OR alzheimer* OR creutzfeldt OR cjd OR huntington* OR binswanger* OR korsako* OR "major neurocognitive disorder" OR "major neurocognitive disorders" ) ) ) OR ( TITLE-ABS-KEY ( ( lewy* W/2 bod* ) ) ) OR ( TITLE-ABS-KEY ( ( pick* W/2 disease ) ) ) OR ( TITLE-ABS-KEY ( ( cerebr* W/2 deteriorat* ) ) ) OR ( TITLE-ABS-KEY ( ( frontotemporal W/2 degenerat* ) ) ) ) | 528,981 |
| --- | --- | --- |
| 2 | ( ( TITLE-ABS-KEY ( ( computer OR automated ) W/2 reasoning ) ) OR ( TITLE-ABS-KEY ( ai OR nlp ) ) OR ( TITLE-ABS-KEY ( "natural language processing" ) ) OR ( TITLE-ABS-KEY ( knowledge W/2 ( acquisition OR representation* ) ) ) OR ( TITLE-ABS-KEY ( computer W/2 ( vision OR knowledge ) ) ) OR ( TITLE-ABS-KEY ( ( artificial OR machine OR deep OR hierarchical OR ambient OR comput* OR assist* ) W/2 ( intelligence OR learning ) ) ) OR ( TITLE-ABS-KEY ( home W/5 ( automat* OR monitor* ) ) ) OR ( TITLE-ABS-KEY ( surveillance OR gps OR track* OR position* OR sensor* OR "big data" OR "open data" OR cloud OR 4g* OR 5g* OR bluetooth OR wearable* OR wireless ) ) OR ( TITLE-ABS-KEY ( "social media" OR "social network" OR "social networks" OR facebook OR twitter OR youtube OR instagram OR flickr OR linkedin OR blog* OR "on-line community" OR "on-line communities" OR "online community" OR "online communities" OR wiki* ) ) OR ( TITLE-ABS-KEY ( "personalized medicine" OR "personalised medicine" OR interoperability OR "decision support" ) ) OR ( TITLE-ABS-KEY ( ( clinical OR medical ) W/2 ( alert OR reminder ) ) ) OR ( TITLE-ABS-KEY ( ( clinical OR medical ) W/2 ( alert OR reminder ) ) ) OR ( TITLE-ABS-KEY ( ( health OR medical OR hospital ) W/2 ( record* OR informati* OR data OR science ) ) ) OR ( TITLE-ABS-KEY ( robot* ) ) OR ( TITLE-ABS-KEY ( gaming OR game OR exergam* ) ) OR ( TITLE-ABS-KEY ( mobile* OR portable OR phone* OR telephone* OR smartphone* OR cellphone* OR smartwatch* OR screen OR touchscreen OR multimedia OR "multi media" ) ) OR ( TITLE-ABS-KEY ( app OR apps ) ) OR ( TITLE-ABS-KEY ( ( internet OR online OR web* OR tele* OR computer OR cyberspace OR "cyber space" OR remote OR virtual OR electronic ) W/2 ( consult* OR based OR application* OR intervention* OR program* OR therap* OR rehabilitat* OR prescri* ) ) ) OR ( TITLE-ABS-KEY ( technolog* OR digital* ) ) OR ( TITLE-ABS-KEY ( ( medical OR biomedical OR health OR clinical ) W/2 informatics ) ) OR ( TITLE-ABS-KEY ( telemedicine OR telehealth OR telemonitoring OR remote AND monitoring ) ) OR ( TITLE-ABS-KEY ( mhealth OR m-health OR ehealth OR e-health OR emental OR e-mental OR e-prescri* ) ) OR ( TITLE-ABS-KEY ( ( medical OR health ) W/2 monitor* ) ) ) | 16,489,948 |
| 3 | ( ( TITLE-ABS-KEY ( ( meta PRE/ analy* ) OR metaanaly* OR meta-analy* ) ) OR ( TITLE-ABS-KEY ( ( systematic OR scoping ) W/2 ( review* OR overview* ) ) ) ) | 773,373 |
| 4 | 1 AND 2 AND 3 | 3,104 |

**Epistemonikos**

| 1 | (title:(dement* OR deliri* OR alzheimer* OR huntington* OR binswanger* OR korsako* OR creutzfeldt OR cjd OR lewy* OR pick* OR "major neurocognitive disorder" OR "major neurocognitive disorders" OR "cerebral deterioration" OR "frontotemporal degeneration") OR abstract:(dement* OR deliri* OR alzheimer* OR huntington* OR binswanger* OR korsako* OR creutzfeldt OR cjd OR lewy* OR pick* OR "major neurocognitive disorder" OR "major neurocognitive disorders" OR "cerebral deterioration" OR "frontotemporal degeneration")) | 46,451 |
| --- | --- | --- |
| 2 | (title:(digital OR technolog* OR telemedicine OR telehealth OR telemonitoring OR "remote monitoring" OR mhealth OR m-health OR ehealth OR "e-health" OR "health technologies" OR emental OR "e-mental" OR e-prescri* OR "medical monitoring" OR "health monitoring" OR "medical informatics" OR "biomedical informatics" OR "health informatics" OR "clinical informatics" OR internet OR app OR apps OR mobile* OR portable OR phone* OR telephone* OR smartphone* OR cellphone* OR smartwatch* OR screen OR touchscreen OR multimedia OR "multi media" OR gaming OR game OR exergam* OR robot* OR "connected devices" OR "smart devices" OR "clinical alert" OR "clinical reminder" OR "medical alert" OR "medical reminder" OR "personalized medicine" OR "personalised medicine" OR interoperability OR "decision support" OR "social media" OR "social network" OR "social networks" OR surveillance OR GPS OR track* OR position* OR sensor* OR "big data" OR "open data" OR cloud OR 4G* OR 5G* OR bluetooth OR wearable* OR wireless OR AI OR NLP OR "artificial intelligence" OR "machine learning" OR "natural language processing" OR "online consultation" OR "online consultations" OR "electronic consultation" OR "electronic consultations" OR "remote consultation" OR "remote consultations" OR "online intervention" OR "online therapy" OR "online rehabilitation" OR "electronic prescription" OR "electronic prescribing") OR abstract:(digital OR technolog* OR telemedicine OR telehealth OR telemonitoring OR "remote monitoring" OR mhealth OR m-health OR ehealth OR "e-health" OR "health technologies" OR emental OR "e-mental" OR e-prescri* OR "medical monitoring" OR "health monitoring" OR "medical informatics" OR "biomedical informatics" OR "health informatics" OR "clinical informatics" OR internet OR app OR apps OR mobile* OR portable OR phone* OR telephone* OR smartphone* OR cellphone* OR smartwatch* OR screen OR touchscreen OR multimedia OR "multi media" OR gaming OR game OR exergam* OR robot* OR "connected devices" OR "smart devices" OR "clinical alert" OR "clinical reminder" OR "medical alert" OR "medical reminder" OR "personalized medicine" OR "personalised medicine" OR interoperability OR "decision support" OR "social media" OR "social network" OR "social networks" OR surveillance OR GPS OR track* OR position* OR sensor* OR "big data" OR "open data" OR cloud OR 4G* OR 5G* OR bluetooth OR wearable* OR wireless OR AI OR NLP OR "artificial intelligence" OR "machine learning" OR "natural language processing" OR "online consultation" OR "online consultations" OR "electronic consultation" OR "electronic consultations" OR "remote consultation" OR "remote consultations" OR "online intervention" OR "online therapy" OR "online rehabilitation" OR "electronic prescription" OR "electronic prescribing")) | 456,804 |
| 3 | (title:(((systematic OR scoping) AND (review* OR overview*)) OR ((meta analy*) OR metaanaly* OR meta-analy*)) OR abstract:(((systematic OR scoping) AND (review* OR overview*)) OR ((meta analy*) OR metaanaly* OR meta-analy*))) | 476,960 |
| 4 | 1 AND 2 AND 3 | 1065 |
