# Supplementary material for: Use of Digital Health Technologies for Dementia Care: Bibliometric Analysis and Report
Source: JMIR Ment Health. 2025 Feb 10;12:e64445. doi: 10.2196/64445 (PMC11851039; doi:10.2196/64445)
Supplement: Multimedia Appendix 3 [file mental_v12i1e64445_app3.docx]

**Multimedia Appendix 3 – Identified overviews or umbrella reviews during the second screening phase**

Digital technologies to prevent falls in people living with dementia or mild cognitive impairment: A rapid systematic overview of systematic reviews - **Wearable technology/sensors, Virtual reality, Environmental sensor-based systems/video systems, Exergaming and commercial games consoles – Low quality**

Ethical and practical concerns of surveillance technologies in residential care for people with dementia or intellectual disabilities: an overview of the literature – **Tracking and GPS systems, intelligent monitoring system – Adequate quality**

Eyes on dementia: an overview of the interplay between eye movements and cognitive decline - **Functional brain imaging techniques, Artificial Intelligence and Machine Learning**

Overview and strategy analysis of technology-based nonpharmacological interventions for in-hospital delirium prevention and reduction: Systematic scoping review – **Not an overview**

Self-Efficacy of Older People Using Technology to Self-Manage COPD, Hypertension, Heart Failure, or Dementia at Home: An Overview of Systematic Reviews – **Telehealth (2 reviews only and not only for Dementia), mHealth, PDA**

**Web-Based** Multidomain Lifestyle Programs for Brain Health: Comprehensive Overview and Meta-Analysis – **mHealth**

An overview of the dyadic, intergenerational and digital-based reminiscence therapy: a scoping review - **Not an overview**

Ethics of using assistive technology in the care for community-dwelling elderly people: an overview of the literature – **Several modalities – Not related to our work**

Efficacy of **virtual reality** technology interventions for cognitive and mental outcomes in older people with cognitive disorders: An umbrella review comprising meta-analyses of randomized controlled trials - **Virtual reality**

Is research on 'smart living environments' based on unobtrusive technologies for older adults going in circles? Evidence from an umbrella review - **“Smart Homes”, “Telemonitoring”, “Ambient Assisted Living”**

**eHealth and Web-Based** Interventions for Informal Carers of People With Dementia in the Community: Umbrella Review - **Including web, telephone, DVD, or a combination of these**

The performance of **artificial intelligence-driven** technologies in diagnosing mental disorders: an umbrella review

Non-pharmacological interventions for neuropsychiatric symptoms of dementia in residential aged care settings: An umbrella review - **Person-tailored interventions, Sensory stimulation, Environmental interventions, Cognitive interventions, Animal-Assisted interventions**

Factors Influencing Implementation of **eHealth Technologies** to Support Informal Dementia Care: Umbrella Review.
