# Supplementary material for: Use of Digital Health Technologies for Dementia Care: Bibliometric Analysis and Report
Source: JMIR Ment Health. 2025 Feb 10;12:e64445. doi: 10.2196/64445 (PMC11851039; doi:10.2196/64445)
Supplement: Multimedia Appendix 5 [file mental_v12i1e64445_app5.docx]

**Multimedia Appendix 5 – References without a digital object identifier**

1. Bernardes MS, Valdrighi JdC, Pereira J, Domingos LB, Santana CdS. Mobile technology for managing health of elderly: a literature review. J health inform. 2016;8(supl.I):1081-8.

2. Carmo EGd, Zazzetta MS, Costa JLR. Robotics in assistance to elderly with Alzheimer's disease: the advantages and intervention of that challenges. Estud interdiscip envelhec. 2016;21(2):47-74.

3. D’Onofrio G, Sancarlo D, Ricciardi F, Ruan Q, Yu Z, Giuliani F, et al. Cognitive stimulation and information-communication technologies (ICT) in Alzheimer’s disease: A systematic review. Nova Science Publishers, Inc.; 2016. p. 1-14.

4. Dyer EA, Kansagara D, McInnes DK, Freeman M, Woods S. Mobile Applications and Internet-based Approaches for Supporting Non-professional Caregivers: A Systematic Review2012.

5. Health Quality O. Prevention of falls and fall-related injuries in community-dwelling seniors: an evidence-based analysis. Ontario health technology assessment series. 2008;8(2):1-78.

6. Mahendra N, Kim ES, Bayles KA, Hopper T, Cleary SJ, Azuma T. Evidence-based practice recommendations for working with individuals with dementia: Computer-assisted cognitive interventions (CACIs). Journal of Medical Speech-Language Pathology. 2005;13(4):xxxv-xliv.

7. Nasir NFBM, Subramaniam P, Ghazali SE, Sharma S, Hassan JM, Lang TH. An Overview Of The Dyadic, Intergenerational And Digital-Based Reminiscence Therapy: A Scoping Review. Malays J Public Health Med. 2023;23(3):119-36.

8. Nemcíková M, Katreniaková Z, Dobríková P, Nagyová I. Burden reduction of informal carers of persons with dementia related to Alzheimer’s disease: A systematic review of effective interventions. Soc Pr. 2020;20(6):122-40.

9. Parra-Vidales E, Soto-Perez F, Perea-Bartolome MV, Franco-Martin MA, Munoz-Sanchez JL. Online interventions for caregivers of people with dementia: a systematic review. Actas espanolas de psiquiatria. 2017;45(3):116-26.

10. Sánchez López MA, Fernández Alemán JL, Toval A, Carrillo de Gea JM. Smart Phones for the elderly: a review of mobile health applications. Rev costarric salud pública. 2015;24(1):30-42.

11. Skinner C, Finkelstein J, editors. Review of mobile phone use in preventive medicine and disease management2008.

12. Zulkifley NH, Manaf RA, Ying LP, Ismail S. Educational intervention for informal caregiver of person with dementia: A systematic review. Malays J Med Health Sci. 2020;16(1):325-31.
