# Supplementary material for: Use of Digital Health Technologies for Dementia Care: Bibliometric Analysis and Report
Source: JMIR Ment Health. 2025 Feb 10;12:e64445. doi: 10.2196/64445 (PMC11851039; doi:10.2196/64445)
Supplement: Multimedia Appendix 6 [file mental_v12i1e64445_app6.docx]

**Multimedia Appendix 6 – References not indexed in Scopus**

1. Zuschnegg J, Schoberer D, Häussl A, Russegger S, Ploder K, Fellner M, et al. Computerized cognitive interventions for preventing dementia in people with mild cognitive impairment: A systematic review with meta-analyses. Alzheimer's & Dementia : the journal of the Alzheimer's Association. 2022;18 Suppl 2:e062179.

2. Mueller KD. A Review of Computer-Based Cognitive Training for Individuals With Mild Cognitive Impairment and Alzheimer's Disease. Perspectives of the ASHA Special Interest Groups. 2016;1(2):47-61.

3. Hodgkinson B, Koch S, Nay R, Lewis M. Managing the wandering behaviour of people living in a residential aged care facility. JBI library of systematic reviews. 2007;5(8):454-96.

4. Ferreira Santana R, Vaqueiro Dantas R, da Silva Soares T, Melo Delphino T, Serra Hercules AB, Teixeira Leite Junior HM. Telecare To Elderly People With Alzheimer And Their Caregivers: Systematic Review. Ciencia, Cuidado e Saude. 2018;17(4):1-6.

5. Blythe SL, Chang E, Johnson A, Griffiths R. The efficacy of nurse implemented non-pharmacological strategies for the symptom management of agitation in persons with advanced dementia living in residential aged care facilities: a systematic review. JBI library of systematic reviews. 2009;7(22):975-1003.

6. Ostaszkiewicz J, Kosowicz L, Cecil J, Somanader D, Dow B. The management of urinary incontinence in nursing homes: a scoping review. Australian & New Zealand Continence Journal. 2023;29(4):80-100.

7. Burton JK, Craig L, Yong SQ, Siddiqi N, Teale EA, Woodhouse R, et al. Non-pharmacological interventions for preventing delirium in hospitalised non-ICU patients. The Cochrane database of systematic reviews. 2021;11((Burton) Academic Geriatric Medicine, Institute of Cardiovascular and Medical Sciences, University of Glasgow, Glasgow, United Kingdom (Craig, Quinn) Institute of Cardiovascular and Medical Sciences, University of Glasgow, Glasgow, United Kingdom(Yong) MVL):CD013307.

8. Sood P, Kletzel S, Negm A, Heyn P, Krishnan S, Devos H. Effectiveness of Brain Gaming in Older Adults with Mild Cognitive Impairment or Dementia: A Systematic Review and Meta-analysis. Archives of Physical Medicine and Rehabilitation. 2020;101(11):e114.

9. Stasiulis E, Naglie G, Sanford S, Belchior P, Crizzle A, Gelinas I, et al. Developing the Driving and Dementia Roadmap: a knowledge-to-action process. International psychogeriatrics. 2023((Stasiulis, Naglie, Sanford) Rotman Research Institute, Baycrest Health Sciences, Toronto, Canada (Stasiulis, Naglie, Sanford) Department of Medicine, Baycrest Health Sciences, Toronto, Canada (Naglie) KITE Research Institute, University Health Network, Tor):1-14.

10. Hopper T, Bourgeois M, Pimentel J, Qualls CD, Hickey E, Frymark T, et al. An evidence-based systematic review on cognitive interventions for individuals with dementia. American Journal of Speech-Language Pathology. 2013;22(1):126-45.

11. Borchert R, Azevedo T, Badhwar A, Bernal J, Betts M, Bruffaerts R, et al. Artificial intelligence for diagnosis and prognosis in neuroimaging for dementia; a systematic review. medRxiv. 2021((Borchert, Malpetti, Peres, Rittman) Department of Clinical Neurosciences, University of Cambridge, Cambridge, United Kingdom (Azevedo) Department of Computer Science and Technology, University of Cambridge, Cambridge, United Kingdom (Badhwar).

12. Eaglestone G, Gkaintatzi E, Stoner C, Pacella R, McCrone P. Effectiveness of community non-pharmacological interventions for mild cognitive impairment and dementia: a systematic review of economic evaluations and a review of reviews. medRxiv. 2022((Eaglestone, Gkaintatzi, Stoner, Pacella, McCrone) Institute for Lifecourse Development, University of Greenwich, United Kingdom).

13. Ciampi A, Rouette J, Pellegrini F, Simoneau G, Caba B, Gafson A, et al. The Use of Machine Learning Methods in Neurodegenerative Disease Research: A Scoping Review. medRxiv. 2023((Ciampi, Rouette) Department of Epidemiology, Biostatistics and Occupational Health, McGill University, Montreal, QC, Canada(Rouette) Centre for Clinical Epidemiology, Lady Davis Institute, Jewish General Hospital, Montreal, QC, Canada(Pellegrini) Biogen).

14. Hitch D, Swan J, Pattison R, Stefaniak R. Use of touchscreen tablet technology by people with dementia in homes: A scoping review. Journal of rehabilitation and assistive technologies engineering. 2017;4(101671667):2055668317733382.

15. Vermeer Y, Higgs P, Charlesworth G. What do we require from surveillance technology? A review of the needs of people with dementia and informal caregivers. Journal of rehabilitation and assistive technologies engineering. 2019;6(101671667):2055668319869517.

16. Tunnard I, Gillam J, Vickerstaff V, Harvey C, Davies N, Ellis-Smith C, et al. The Acceptability and Effectiveness of eHealth Interventions to Support Assessment and Decision Making for People With Dementia Living in Care Homes: A Systematic Review. Palliative Medicine. 2022;36(1 SUPPL):113.

17. Riley CO, McKinstry B, Fairhurst K. Accuracy of telephone screening tools to identify dementia patients remotely: systematic review. JRSM Open. 2022;13(9).

18. Appel L, Ali S, Narag T, Mozeson K, Pasat Z, Orchanian-Cheff A, et al. Virtual reality to promote wellbeing in persons with dementia: A scoping review. Journal of rehabilitation and assistive technologies engineering. 2021;8(101671667):20556683211053952.

19. Hung L, Wong J, Smith C, Berndt A, Gregorio M, Horne N, et al. Facilitators and barriers to using telepresence robots in aged care settings: A scoping review. Journal of rehabilitation and assistive technologies engineering. 2022;9(101671667):20556683211072385.

20. Anderson M, Menon R, Oak K, Allan L. The use of technology for social interaction by people with dementia: A scoping review. PLOS digital health. 2022;1(6):e0000053.

21. Koo BM, Vizer LM. Examining Mobile Technologies to Support Older Adults With Dementia Through the Lens of Personhood and Human Needs: Scoping Review. JMIR mHealth and uHealth. 2019;7(11):e15122.

22. Rieckmann N, Schwarzbach C, Nocon M, Roll S, Vauth C, Willich SN, et al. Concepts of care for people with dementia. GMS health technology assessment. 2009;5(101276045):Doc01.

23. David L, Popa SL, Barsan M, Muresan L, Ismaiel A, Popa LC, et al. Nursing procedures for advanced dementia: Traditional techniques versus autonomous robotic applications (Review). Experimental and therapeutic medicine. 2022;23(2):124.

24. Rueda Diaz LJ, Da Monteiro Cruz DL. The efficacy of telephone use to assist and improve the wellbeing of family caregivers of persons with chronic diseases: A systematic review. JBI Database of Systematic Reviews and Implementation Reports. 2014;12(12):106-40.

25. Travers C, Brooks D, Hines S, O'Reilly M, McMaster M, He W, et al. Effectiveness of meaningful occupation interventions for people living with dementia in residential aged care: A systematic review. JBI Database of Systematic Reviews and Implementation Reports. 2016;14(12):163-225.

26. Bagheri N, Wangdi K, Cherbuin N, Anstey KJ. Combining Geospatial Analysis with Dementia Risk Utilising General Practice Data: A Systematic Review. The journal of prevention of Alzheimer's disease. 2018;5(1):71-7.

27. Kalimisetty S, Askar W, Fay B, Khan A. Models for Predicting Incident Delirium in Hospitalized Older Adults: A Systematic Review. Journal of patient-centered research and reviews. 2017;4(2):69-77.

28. Elfaki AO, Alotaibi M. The role of M-health applications in the fight against Alzheimer's: current and future directions. mHealth. 2018;4(101678564):32
